# Isomeric decker metallo-supramolecules with tunable luminescence and chiroptical properties

**DOI:** 10.1039/d5sc04596g

**Published:** 2025-10-03

**Authors:** Ningxu Han, Jianjun Ma, Hao Yu, Junjuan Shi, Manman Dai, Ziteng Guo, Zinuo Gao, Houyu Zhang, Ming Wang

**Affiliations:** a State Key Laboratory of Supramolecular Structure and Materials, College of Chemistry, Jilin University Changchun Jilin 130012 China mingwang358@jlu.edu.cn houyuzhang@jlu.edu.cn; b Engineering Research Center of Xinjiang and Central Asian Medicine Resources, Ministry of Education, Xinjiang Medical University Urumqi Xinjiang 830011 China

## Abstract

The regulation of interchromophoric interactions in multichromophoric systems is crucial for developing high-performance photofunctional materials. In this study, we present a novel self-assembly strategy to construct isomeric decker complexes, denoted as S1 and S2, which integrate disparate chromophores, achiral BODIPY and chiral binaphthyl moieties. This isomerization results in distinct chromophore packing modes. In the case of S1, the BODIPY and binaphthyl moieties are arranged in a relatively loose manner (∼6.0 Å), enabling efficient FRET and preserving the strong locally excited (LE) emission (*Φ*_F_ = 91.3%) characteristic of the BODIPY unit. In contrast, for S2, the denser packing between the BODIPY and binaphthyl moieties (∼4.8 Å) leads to through-space charge transfer (TSCT) and weak charge transfer (CT) emissions (*Φ*_F_ = 8.6%). Notably, only complex (R)/(S)-S1 shows mirror-image circular dichroism (CD) signals based on chirality transfer and circularly polarized luminescence (CPL), as supported by TD-DFT calculations, which reveal that the binaphthyl moiety alters the angle between the electric transition dipole moment (μ) and the magnetic transition dipole moment (*m*).

## Abstract 2

This work presents a novel self-assembly strategy for constructing isomeric decker metallo-supramolecules. The subtle variation in chromophore arrangement results in distinct luminescence and chiroptical properties.

## Graphical abstract

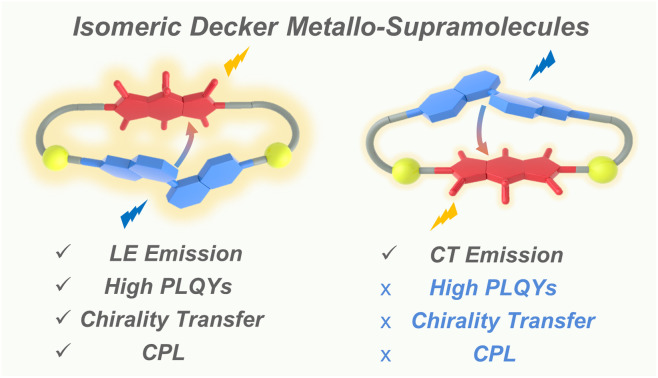

## Introduction

The interplay between chromophores is pivotal in nature, as exemplified by the excitation energy transfer (EET) observed among chromophores such as chlorophylls, carotenoids, and phycobilins in photosynthetic organisms.^1^ These chromophores can be organized into particular arrangements by the surrounding protein scaffolds, resulting in modulated excitonic interactions that optimize the light-harvesting properties and EET efficiency, crucial for transforming solar energy into chemical energy.^2^ Inspired by nature, researchers realize that the fine-tuning of chromophore arrangements is crucial for efficient interchromophoric interactions, thereby improving material performance in relation to light-harvesting,^3–7^ emission,^8–12^ and chiral fields.^13–16^ In this context, artificial synthetic skeletons capable of manipulating the chromophore arrangements have garnered increasing attention. In multichromophoric systems, many through-space interactions, such as EET,^17–20^ through-space charge transfer (TSCT),^21–24^ and chirality transfer,^25–27^ are mainly governed by specific spatial orientations and distance between chromophores. For instance, efficient energy transfer between donor and acceptor chromophores typically requires a distance of less than 10 nm due to dipole–dipole coupling.^28,29^ When an electron-rich donor and an electron-deficient acceptor are positioned in a close face-to-face manner, with a spatial distance of ∼4 Å, a CT interaction can occur and lead to a new excited state with a narrowed energy gap. Notably, when a chiral chromophore is strategically oriented relative to an achiral counterpart, it can establish a chiral microenvironment around the achiral molecules, facilitating chirality transfer and inducing their chiroptical properties. Currently, significant efforts are being devoted to exploring suitable synthetic skeletons capable of precisely regulating chromophore arrangements, thereby advancing the understanding of structure–performance relationships.

**Fig. 1:**
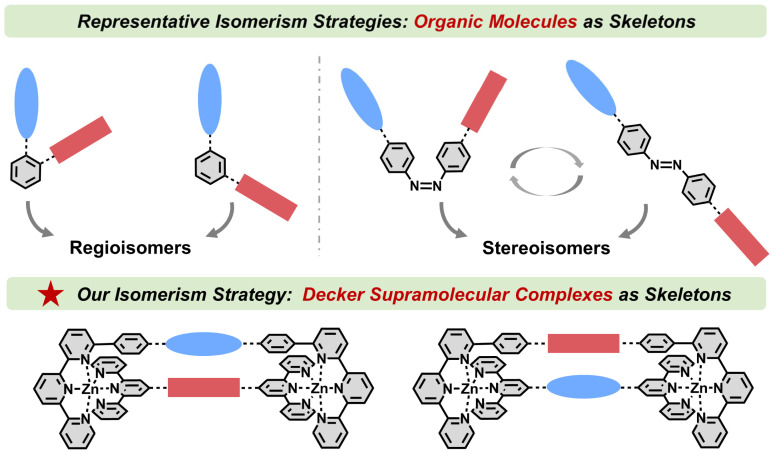
Examples of previous isomerism strategies and our proposed isomerism strategy based on decker supramolecular skeletons in this work.

Isomerism of photofunctional molecules represents a powerful strategy for precisely controlling the spatial arrangement of functional groups, thereby optimizing material performance. For organic molecules, common strategies to achieve isomerism (Fig. 1) involve either modifying identical chromophore or substituent pairs at distinct positions within the same molecular skeleton (regioisomerism),^30–34^ or utilizing photoswitches as molecular skeletons to facilitate *trans*-to-*cis* isomerization (stereoisomerism).^35–40^ For example, Tang *et al.* regulated the packing mode and solid-state fluorescence of isomeric molecules by tuning the substituted positions of chromophores.^41^ Furthermore, the isomerism strategy can be employed to tailor organic ligands, enabling the facile synthesis of isomeric metallo-supramolecules^42–46^ or metal–organic frameworks.^47–50^ For example, Nitschke *et al.* prepared isomeric coordination cages by employing isomeric anthracene-based ligands and studied their stimuli-driven guest uptake and release.^51^ These innovative strategies inspire the modulation of chromophore arrangements and properties through the application of isomerism. However, examples demonstrating modulation of through-space chromophore interactions *via* isomerism-driven subtle adjustments of chromophore arrangements remain limited. This could be attributed to the following two reasons: (1) the challenges in designing molecular skeletons capable of simultaneously achieving isomerism and effective through-space interactions and (2) rigid interchromophoric interactions that are resistant to modulation through subtle alterations in spatial arrangements.

**Fig. 2:**
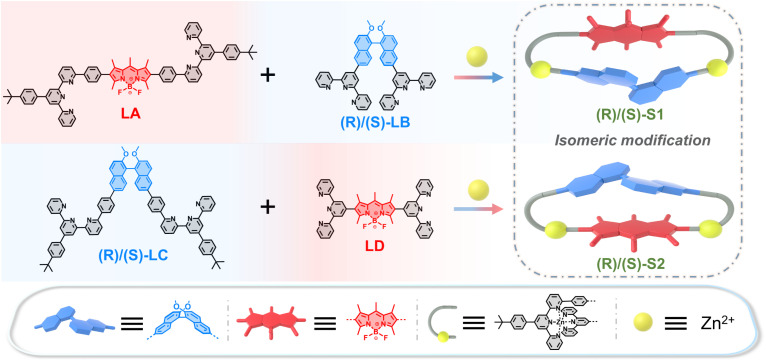
Design and self-assembly of ligands and isomeric decker complexes.

Herein, we propose a novel design strategy to realize the regioisomerism of heterochromophore decker complexes S1 and S2 with well-defined geometry (Fig. 2). Two types of functional chromophores, boron-dipyrromethene (BODIPY) and binaphthyl moieties, are incorporated into each of the complexes. By employing dissymmetrical modified 2,2′:6′,2′′-terpyridine (tpy) units, two chromophores can be precisely placed in close spatial proximity, which provides a suitable model for the comparative study of interchromophoric interactions in isomeric systems. It is impressive that these two isomeric decker complexes show completely different photophysical processes, confirmed by photophysical characterization and DFT and TD-DFT calculations. Complex S1 exhibits outstanding LE emission, while complex S2 shows quenched CT emission. Additionally, compared with significant chirality transfer from LB to LA in S1, no similar phenomenon is observed in S2. These findings demonstrate that our decker supramolecular skeleton enables isomeric modification and adjustable interactions between chromophores, offering significant insights for the development of high-performance emissive and chiral materials.

## Results and discussion

### Synthesis and characterization of heterochromophore decker complexes

We designed and synthesized four tpy ligands, in which LA and LD contain a BODIPY moiety and enantiopure ligands LB (ref. 52) and LC contain a chiral binaphthyl moiety, all in decent yields. The synthetic procedures and full characterization by ^1^H and ^13^C nuclear magnetic resonance (NMR) spectroscopy, two-dimensional correlation spectroscopy (2D-COSY), and matrix-assisted laser desorption/ionization time-of-flight (MALDI-TOF) mass spectrometry of all ligands are shown in the SI (Fig. S1 and S4–S18). Isomeric complexes S1 and S2 were assembled by mixing the ditopic ligands LA and LB for S1, and ligands LC and LD for S2, respectively, with Zn(NO_3_)_2_·6H_2_O in exact stoichiometric ratios of 1 : 1 : 2 in CHCl_3_/MeOH at 50 °C for 10 h. Following the excess addition of NH_4_PF_6_, red complexes were obtained without further purification.

**Fig. 3:**
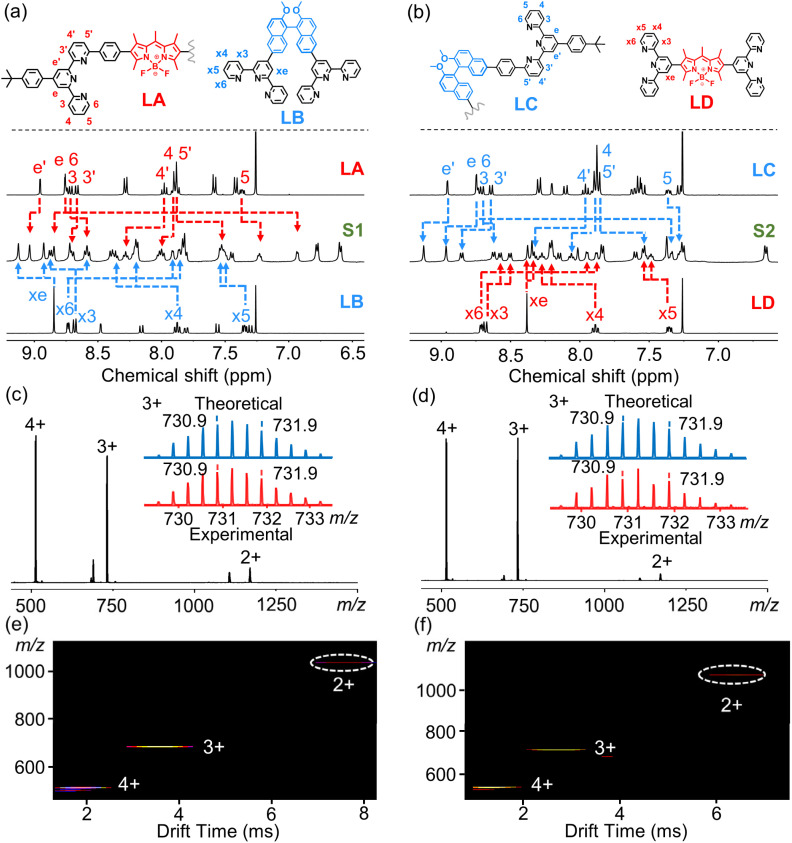
^1^H NMR spectra (400 MHz, 298 K) of (a) ligands LA and LB in CDCl_3_ and complex S1 in CD_3_CN and (b) ligands LC and LD in CDCl_3_ and complex S2 in CD_3_CN. ESI-MS spectra of complexes (c) S1 and (d) S2. TWIM-MS spectra of complexes (e) S1 and (f) S2.

NMR spectroscopy was employed to confirm the generation of the complexes. As shown in Fig. 3a and b, ^1^H-NMR spectra of these two complexes exhibited sharp and well-resolved proton signals, indicating the probable construction of discrete assemblies. NMR resonances of complexes were further assigned by 2D-COSY and nuclear Overhauser effect spectroscopy (NOESY) (Fig. S19–S30). In contrast to the rigid, linearly configured BODIPY-based ligands, LB and LC showed flexible geometries, resulting from axially chiral binaphthyl cores. Upon complexation, the conformations of both LB and LC were strongly rigidified, resulting in a distinct chemical environment of the tpy protons within each ligand. Through accurate integration analysis, the stoichiometries of different ligands in the complexes were proved to be 1 : 1, as expected for isomeric complexes. Diffusion-ordered NMR spectroscopy (DOSY) showed that all the protons of each complex had the same band (Fig. S33 and S34), consistent with discrete assemblies with a single component. Electrospray ionization mass spectrometry (ESI-MS) showed a set of prominent peaks arising from consecutive charge states (2+ to 4+) due to the loss of anions (PF_6_^−^), consistent with the expected heteroleptic complexes (Fig. 3c and d). In addition, the experimental isotope patterns of each charge state matched well with their simulated counterparts (Fig. S2 and S3). Traveling wave ion mobility-mass spectrometry (TWIM-MS) displayed the narrow drift time distributions of each charge state for the complexes (Fig. 3e and f), indicative of the formation of a single product.

**Fig. 4:**
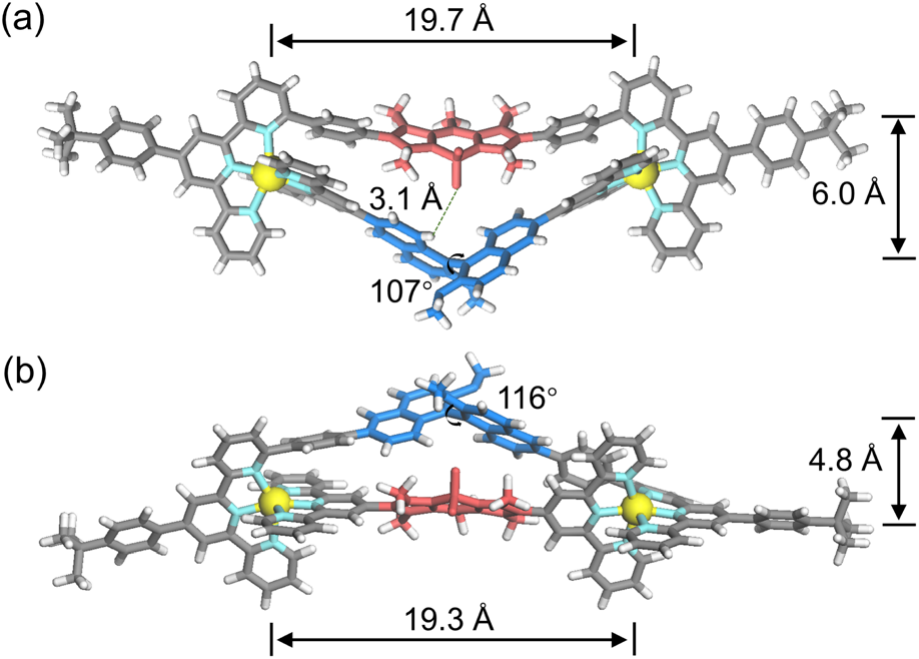
(a) X-ray crystal structure of complex (R)-S1. (b) DFT model of (S)-S2. All solvent molecules and counterions are omitted for clarity.

A single crystal of complex (R)-S1 was obtained by slow vapor diffusion of ethyl acetate into the MeCN solution of complex (R)-S1 over a period of 4 weeks. The single-crystal structure of complex S1 validated the precision of our heteroleptic self-assembly strategy, where ligand LA with six-position-modified tpy units selectively bound ligand LB based on shape complementary assembly (Fig. 4a).^53,54^ To accommodate the rigid ligand LA with a fixed arm length, the axially chiral binaphthyl core with adaptable configuration adopted a specific twist, where the dihedral angle of two different naphthyl planes was ∼107°. The methoxy groups adopted the orientation outward the sterically crowded triangular cavity of complex S1. The C–H⋯F hydrogen bond interactions (∼3.1 Å) were observed between binaphthyl protons and fluorine atoms in BODIPY units. A DFT-optimized model of S2 is shown in Fig. 4b, and it exhibited a more congested triangular cavity, as evidenced by the reduced Zn⋯Zn distance (∼19.3 Å) and interchromophoric distance (∼4.8 Å) compared to those in S1 (∼19.7 and 6.0 Å, respectively).

### Photophysical properties

The photophysical properties of all ligands and complexes in solution were investigated using UV–vis absorption and fluorescence spectroscopy. The absorption spectra of ligands LB and LC (10 μM in CHCl_3_) exhibited broad bands between 250 and 375 nm (Fig. S36a). In contrast, ligands LA and LD displayed additional absorption bands in the range of 450–580 nm (Fig. S35a), centered at 529 nm and 521 nm, respectively, which mainly originated from the electronic transitions of BODIPY moieties. To further understand the photophysical properties of ligands, we performed quantum-chemical calculations using DFT and TD-DFT methods. In ligands LA and LD, the highest occupied molecular orbitals (HOMOs) and the lowest unoccupied molecular orbitals (LUMOs) are mainly located on the BODIPY moieties (Fig. S50), indicative of negligible CT character. The lowest-energy absorption bands of ligands LA and LD are mainly ascribed to the HOMO → LUMO transitions, with high oscillator strengths (*f*) of 1.26 and 1.18, respectively. For ligands LB and LC, the HOMOs are mainly located on the binaphthyl units, whereas the LUMOs are concentrated on the tpy moieties (Fig. S51). The lowest-energy absorption bands of ligands LB and LC arise mainly from HOMO → LUMO and HOMO → LUMO+2 transitions (Fig. S51), with corresponding oscillator strengths of 0.65 and 0.60, respectively.

The photoluminescence (PL) spectra of all the ligands (Fig. S35b and S36b) exhibited intense emission bands with maximum emission wavelengths at 573 nm, 409 nm, 396 nm, and 546 nm for LA, LB, LC, and LD, respectively. Their photoluminescence quantum yields (*Φ*_F_) and lifetimes in CHCl_3_ are determined as follows: 99.7% and 4.37 ns for LA, 51.6% and 4.37 ns for LB, 67.0% and 1.53 ns for LC, and 99.8% and 4.42 ns for LD, respectively (Fig. S46, S47 and Table S1). For binaphthyl-based ligands LB and LC, pronounced solvatochromism behavior was observed (Fig. S38 and S39), as a result of the CT transition from binaphthyl to tpy moieties. On the other hand, such a phenomenon was absent for BODIPY-based ligands LA and LD (Fig. S37 and S40), indicative of their weak CT character. Despite the different coordination units (six-position-modified or unmodified tpy) attached to the BODIPY moiety, both ligands LA and LD show exceptional photoluminescence quantum yields in CHCl_3_, indicative of the negligible perturbation of these substituents to the intrinsic emissive properties of the BODIPY moiety.

**Fig. 5:**
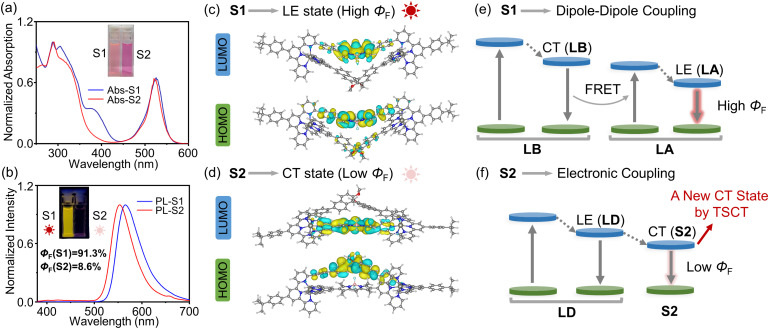
(a) Absorption and (b) fluorescent emission spectra (*λ*_ex_ = 330 nm) of complexes S1 and S2 at the concentration of 10 μM in CH_3_CN. Insets: photographs of complexes S1 and S2 in CH_3_CN under visible light (a) and under 365 nm UV light (b). Molecular orbitals of complexes (c) (R)-S1 and (d) (S)-S2 calculated using TD-DFT. Comparison of proposed photophysical processes in complexes (e) S1 and (f) S2.

For complexes, S1 and S2 exhibited broad absorption bands between 250 and 440 nm and a strong absorption peak at 525 nm for S1 and 522 nm for S2 in CH_3_CN, respectively (Fig. 5a). Despite containing two different ligands, both complexes exhibited single emission bands in CH_3_CN (10 μM; *λ*_ex_ = 330 nm), with maximum emission wavelengths at 564 nm for S1 and 554 nm for S2 (Fig. 5b), respectively, attributed to the strong interplay between different ligands. Similar to highly emissive ligand LA, complex S1 also showed excellent fluorescent properties in CH_3_CN (*Φ*_F_ = 91.3%). However, complex S2 exhibited dramatically quenched emission with a *Φ*_F_ of 8.6% in CH_3_CN, notably lower than the high photoluminescence quantum yield of ligand LD. The pronounced disparity between the isomeric complexes S1 and S2 is tentatively attributed to the different through-space chromophore interactions.

To enable a comparative study between ligands and complexes, we investigated the optical properties of the ligands and complexes (NO_3_^−^ as counterions) in the mixed solvent of CHCl_3_/MeOH (v : v = 1 : 1), due to the good solubility of all the ligands and complexes (Fig. S41–S43). Both ligand LA and complex S1 exhibited comparable absorption peaks between 470 and 600 nm and emission peaks, suggesting the nearly preserved electronic transition of ligand LA upon complexation. The perfect spectral overlap between LB emission and LA absorption bands (Fig. S43) facilitates efficient Förster resonance energy transfer (FRET), as evidenced by the dominant emission peak at 565 nm upon 330 nm excitation. In contrast, complex S2 exhibited a broadened and red-shifted absorption band compared to ligand LD, accompanied by a corresponding red-shifted emission maximum at 557 nm. This spectral behavior was recapitulated in CH_3_CN (Fig. S44 and S45), collectively supporting the hypothesis that through-space interactions within S2 generate a new lowest-lying electronic state.

To elucidate the different photophysical behaviors of isomeric S1 and S2, we performed quantum-chemical calculations using DFT and TD-DFT methods. In complex S1, the HOMOs and LUMOs are mainly located on the BODIPY moieties within ligand LA (Fig. 5c), suggesting negligible CT interactions between chromophores. The lowest-energy absorption band of complex S1 is mainly attributed to the HOMO → LUMO transitions with a high oscillator strength of 0.93 (Fig. S52). Consequently, the highly emissive character of complex S1 can be attributed to the LE state within ligand LA, with the binaphthyl moiety exerting minimal effect on LA's electronic state. Conversely, the HOMOs and LUMOs of complex S2 are mainly concentrated on binaphthyl and BODIPY moieties (Fig. 5d), respectively, indicating that the TSCT from the binaphthyl donor to the BODIPY acceptor plays a significant role in the photophysical processes. Notably, the HOMO → LUMO transitions of S2 exhibit extremely low oscillator strengths (*f* <0.01), resulting in a negligible CT band in the absorption spectrum. The HOMO-2 → LUMO transitions with a high oscillator strength of 1.17 could account for the intense absorption peak at 522 nm (Fig. S53). However, this newly emerged lowest-lying electronic state, associated with the CT state, could have a significant impact on the emission process and explain the substantial reduction in PL quantum yields of complex S2 relative to ligand LD.

The very different through-space interactions within isomeric complexes may originate from the varying chromophore arrangements and their attached skeleton molecules. The chromophore pair in complex S2 exhibits a denser arrangement. As a result, the electronic coupling between binaphthyl and BODIPY moieties in complex S2 is promoted and leads to a new CT state (Fig. 5f). On the other hand, the electron-deficient tpy moieties, directly attached to the BODIPY acceptor in ligand LD, could reduce the electron density on BODIPY, thereby facilitating TSCT in complex S2. Whereas in isomeric complex S1, the tpy moieties were attached to electron-rich binaphthyl moieties, favoring dominant through-bond CT from binaphthyl to tpy moieties over TSCT. In addition, ligand LB, operating as a singular molecular entity, can behave as an energy donor in the FRET process of complex S1 (Fig. 5e).

### Chiroptical properties

**Fig. 6:**
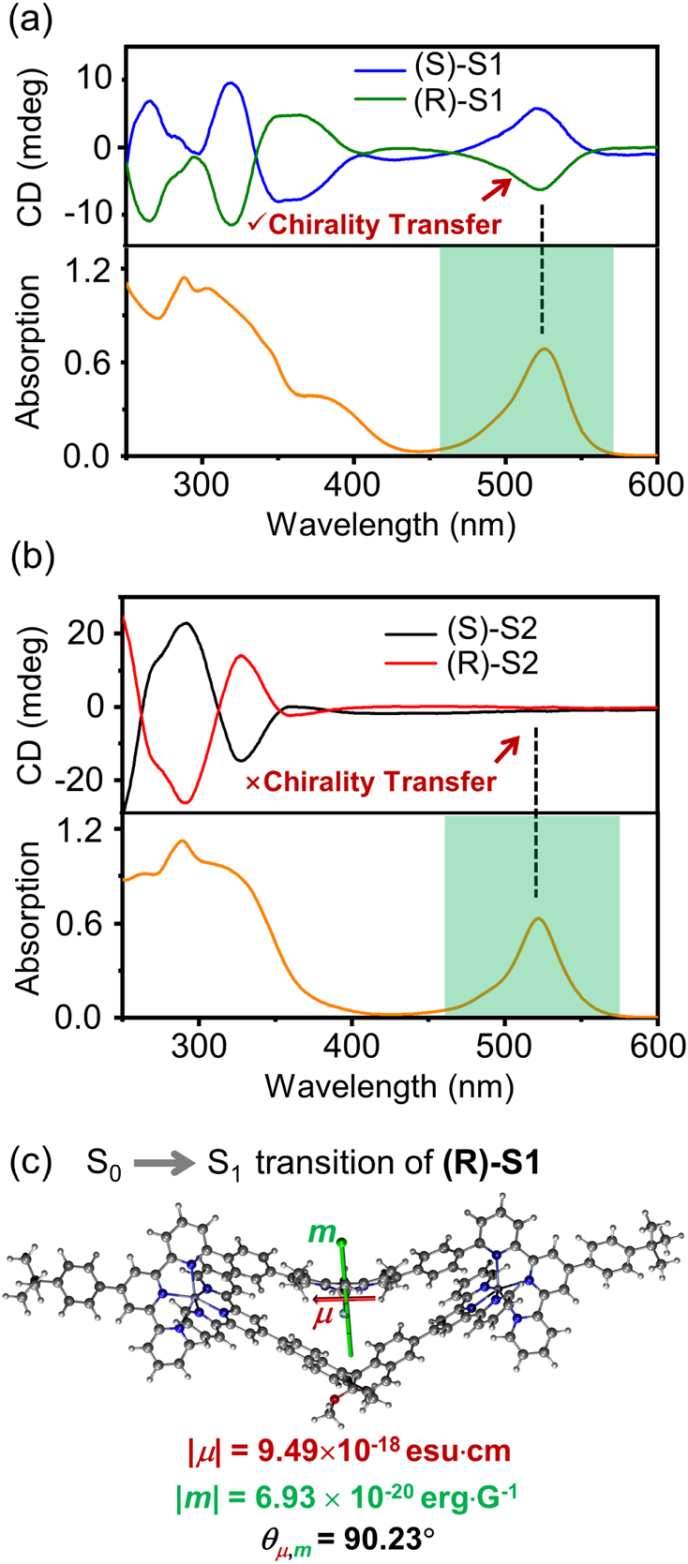
CD and absorption spectra (*c* = 10 μM) of complexes (a) (R)/(S)-S1 and (b) (R)/(S)-S2 in CH_3_CN. (c) S_0_ → S_1_ transition electric and magnetic dipole moments of (R)-S1. The transition magnetic dipole moment vector is shown in green and the transition electric dipole moment vector is shown in red.

The chiroptical properties of complexes S1 and S2 were further investigated by CD spectroscopy to explore the interactions between chiral ligands (LB or LC) and achiral ligands (LA or LD). The mirror-imaged CD signals were observed for (R)/(S)-S1 and (R)/(S)-S2, corresponding to two pairs of enantiomers (Fig. 6a and b). Specifically, complex (R)/(S)-S1 showed a CD signal between 460 and 550 nm, originating from the achiral ligand LA with the BODIPY chromophore, demonstrating the successful chirality transfer from the binaphthyl moiety to the BODIPY chromophore. This is in stark contrast to the silent CD signals of (R)/(S)-S2 in this spectral range.

The chirality transfer observed in complex (R)/(S)-S1 inspires us to explore the interaction mechanism by TD-DFT calculations. According to the theory, the dissymmetry factor for CD (*g*_abs_) is determined by the transition electric dipole moment (*μ*), magnetic dipole moment (*m*), and the angle (*θ*) *via* the simplified equation *g*_abs_ = 4 (|*m*|/|*μ*|)cos *θ*.^55,56^ According to the calculations of complex S1, the absorption band in this range could be assigned to S_0_ → S_1_ transition associated with HOMO → LUMO transition. The HOMOs and LUMOs are mainly located on the BODIPY moieties within ligand LA, indicating minimal involvement of the binaphthyl moiety in this absorption feature. The *μ* and *m* as well as the angle between *μ* and *m* of (R)-S1 for S_0_ → S_1_ transitions were determined using calculations (Fig. 6c). It was found that the angle (*θ* = 90.23°) has changed, deviating from 90°, leading to a negative cos *θ* value (∼−0.004). This theoretical finding correlates well with the experimentally observed negative CD signal for (R)-S1.

**Fig. 7:**
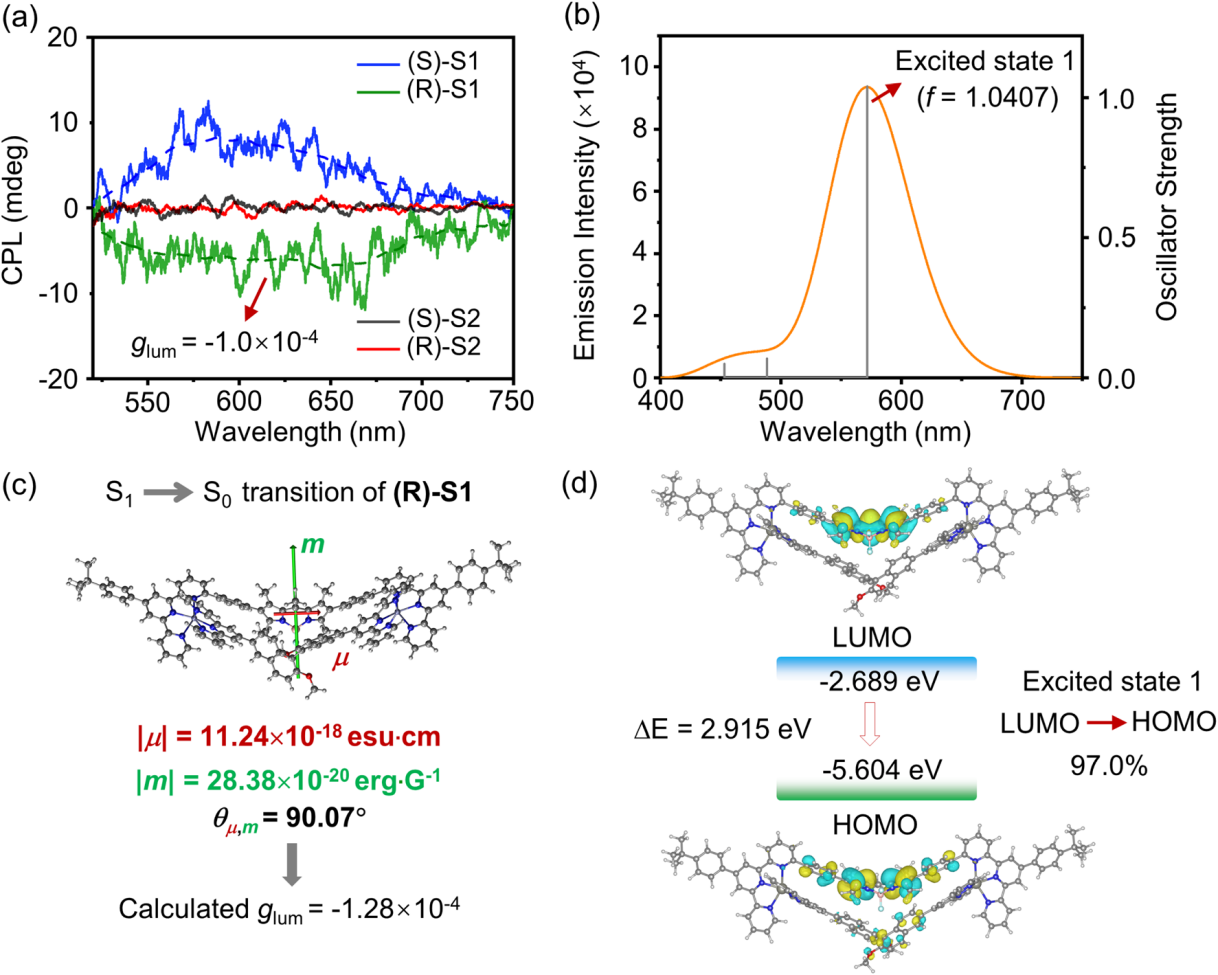
(a) CPL spectra (*c* = 50 μM; *λ*_ex_ = 420 nm) of complexes (R)/(S)-S1 and (R)/(S)-S2 in CH_3_CN. (b) TD-DFT calculated emission spectra of (R)-S1 in CH_3_CN. (c) S_1_ → S_0_ transition electric and magnetic dipole moments of (R)-S1. The transition magnetic dipole moment vector is shown in green and the transition electric dipole moment vector is shown in red. (d) Molecular orbitals of complex (R)-S1 in the excited states calculated using TD-DFT.

To further validate the difference in chirality transfer behaviors of isomeric S1 and S2, the CPL spectra of enantiomers (R)/(S)-S1 and (R)/(S)-S2 were measured in CH_3_CN. As anticipated, (R)/(S)-S1 exhibited mirror-image CPL signals, consistent with the chirality transfer properties observed from CD spectra, whereas (R)/(S)-S2 showed no detectable CPL activity (Fig. 7a). According to the TD-DFT computational results, the HOMOs and LUMOs in the excited state are similar to those in the ground state (Fig. 7d), primarily localized on the BODIPY moiety, which effectively explains the nature of LE emission of complex S1. Furthermore, the first excited state (S_1_ → S_0_) is predominantly contributed by the LUMO → HOMO transition (97%) and exhibits the highest oscillator strength (*f* = 1.0407) (Fig. 7b), significantly higher than that of the second excited state (S_2_ → S_0_). This further confirms that the main fluorescence and circularly polarized luminescence observed experimentally originate from the first excited state (S_1_ → S_0_). Based on these findings, we further calculated *m*, *μ*, and *θ* for the first excited state. Using these values, a calculated *g*_lum_ value of −1.28 × 10^−4^ for (R)-S1 was obtained (Fig. 7c), which is in agreement with the experimental result (−1.0 × 10^−4^).

This chirality transfer property likely arises from the specific spatial arrangements between the binaphthyl and BODIPY moieties, encompassing factors such as spatial distance and relative orientation.^57–59^ Unlike fluorescent groups with inherent chiral conformations, such as tetraphenylethylene^60–62^ and perylene diimide,^63–65^ whose chirality transfer mechanisms rely on the control of their chiral conformations, the crystal structure of (R)-S1 reveals that the BODIPY moiety in ligand LA does not adopt a chiral conformation. This chirality transfer is likely attributed to the chiral microenvironment created by the binaphthyl moiety, which results in an altered angle between *μ* and *m*. Despite the closer proximity of the identical chromophore pair in S2, the absence of chirality transfer underscores the critical role of specific spatial orientation over mere proximity.

## Conclusions

In summary, we propose a novel design strategy for engineering regioisomeric decker metallo-supramolecules with modulated interchromophoric interactions. The identical functional chromophore pair, binaphthyl and BODIPY moieties, is systematically introduced into the regioisomeric decker complexes (R)/(S)-S1 and (R)/(S)-S2. The dense spatial arrangements of chromophores enable efficient interchromophoric interactions, specifically FRET in S1 and TSCT in S2, as evidenced by integrated photophysical characterization and DFT and TD-DFT calculations. We attribute the different interactions to both the distinct chromophore arrangements and the conjugated skeleton molecules. S2 exhibits a denser chromophore arrangement than S1, promoting the electronic coupling. Whereas the skeleton molecules (tpy and benzene) might also affect the electronic nature of the chromophores and their interactions. Furthermore, CD and CPL spectra confirmed the chirality transfer from the chiral binaphthyl to BODIPY moiety within complex S1, a phenomenon not observed in S2 with similar chromophore arrangements. This successful chirality transfer in S1 was unambiguously demonstrated by calculations. We attribute the chiral signal of the achiral BODIPY moiety to the chiral microenvironment created by the binaphthyl group, leading to changes in the angle between *μ* and *m*. This work exemplifies a unique case illustrating how isomeric modification can induce substantial variations in interchromophoric interactions, offering novel insights for the design of advanced photofunctional and chiroptical materials. Furthermore, the modular architecture of this system suggests promising potential for integrating diverse functional units, including luminogens, photoswitches, catalytic centers, radicals, therapeutic agents, or chiral groups, demonstrating its potential for broad applications across multiple disciplines.

## Author contributions

M. W. conceived the study and designed the experiments. N. H., J. M., and J. S. conducted the experiments and analyzed the data. H. Y. collected the X-ray data and refined the structures. M. D., Z. G. and Z. G. performed the ESI-MS and TWIM-MS experiments. H. Z. conducted the DFT calculations. N. H., J. S., H. Z., and M. W. wrote the manuscript. All authors discussed the results and commented on the manuscript.

## Conflicts of interest

There are no conflicts to declare.

## Supplementary Material

SC-016-D5SC04596G-s001

SC-016-D5SC04596G-s002

## Data Availability

The data supporting this article have been included as part of the Supplementary Information (SI). CCDC 2440691 contains the supplementary crystallographic data for this paper.^66^ Supplementary information: synthesis of the ligands and complexes, NMR spectra, MALDI-TOF spectra, ESI-MS data, single-crystal data, absorption spectra, fluorescent emission spectra, CD spectra, and CPL spectra. See DOI: https://doi.org/10.1039/d5sc04596g.
