# Supplementary material for: Isomeric decker metallo-supramolecules with tunable luminescence and chiroptical properties
Source: Chem Sci. 2025 Oct 3;16(44):21010–9. doi: 10.1039/d5sc04596g (PMC12516762; doi:10.1039/d5sc04596g)
Supplement: SC-016-D5SC04596G-s001 [file SC-016-D5SC04596G-s001.pdf]

Supplementary Information (SI) for Chemical Science.
This journal is © The Royal Society of Chemistry 2025

Supplementary information

Isomeric decker metallo-supramolecules with tunable luminescence and chiroptical properties

Ningxu Han,[1] Jianjun Ma,[2] Hao Yu,[1] Junjuan Shi,[1] Manman Dai,[1] Ziteng Guo,[1] Zinuo Gao,[1] Houyu Zhang,[1,*] and Ming Wang[1,*]

[1]State Key Laboratory of Supramolecular Structure and Materials, College of Chemistry, Jilin University, Changchun, Jilin 130012, China

[2] Engineering Research Center of Xinjiang and Central Asian Medicine Resources, Ministry of Education, Xinjiang Medical University, Urumqi, Xinjiang 830011, China

[*]Correspondence and requests for materials should be addressed to
Email: mingwang358@jlu.edu.cn; houyuzhang@jlu.edu.cn

1. Experimental section

General Procedures. All reagents were purchased from Macklin Reagent, Energy chemical, Alfa Aesar and used without further purification. 4'-bromo-2,2':6',2''-terpyridine was purchased from Jilin Chinese Academy of Sciences - Yanshen Technology Co. Ltd. Column chromatography was conducted using SiO_2 (Qingdao ocean column chromatography silica gel, 200-300 mesh) and the separated products were visualized by UV light.

Nuclear magnetic resonance (NMR). NMR spectra data were recorded on Qone AS 400 MHz, Bruker 500 MHz, and 600 MHz Advance NMR spectrometer in $CDCl_3$ or CD_3CN with TMS as reference.

Electrospray ionization-mass spectrometry (ESI-MS) and **travelling wave ion mobility-mass spectrometry (TWIM-MS)**. Electrospray ionization (ESI) mass spectra was recorded with a Waters Synapt G2 tandem mass spectrometer, using solutions of 0.5 mg sample in 1 mL of MeCN/MeOH (3:1, v/v) for complexes. The TWIM-MS experiments were performed under the following conditions: ESI capillary voltage, 3 kV; sample cone voltage, 30 V; extraction cone voltage, 3.5 V; source temperature 100 ºC; desolvation temperature, 100 ºC; cone gas flow, 10 L/h; desolvation gas flow, 700 L/h (N_2); source gas control, 0 mL/min; trap gas control, 2 mL/min; helium cell gas control, 100 mL/min; ion mobility (IM) cell gas control, 30 mL/min; sample flow rate, 5 μL/min; IM traveling wave height, 25 V; and IM traveling wave velocity, 1000 m/s.

Matrix-assisted laser desorption/ionization time-of-flight (MALDI-TOF) mass spectrometry. MALDI-TOF-MS was performed on a Bruker AutoflexIII using trans-2-[3-(4-*tert*-butylphenyl)-2-methyl-2-2-propenyli-dene]malononitrile (DCTB) as a matrix. The matrix dissolved in $CHCl_3$ at 20 mg mL^{-1} and organic compounds were dissolved in $CHCl_3$ at 10 mg mL^{-1}. Sample was prepared by depositing 0.5 μL of matrix on the wells of a 384-well ground-steel plate, allowing the spots to dry, depositing 0.5 μL of the sample on a spot of dry matrix, and adding another 0.5 μL of matrix on top of the dry sample. The plate was inserted into the MALDI source after drying. The

sample was conducted in linear mode. And the data analysis was conducted with Bruker's FlexAnalysis software.

Elemental analyses. Elemental analyses (carbon, hydrogen, nitrogen, sulfur) were performed on the elemental analyzer Elemental Vario EL cube.

Photophysical measurement. UV-vis spectra of solutions were recorded on a PerkinElmer LAMBDA 365 Spectrophotometer. Fluorescence emission spectra was measured by a Shimadzu spectrofluorimeter RF-5301PC. Absolute PL quantum yields, and fluorescence lifetimes were carried out with FLS 980 Spectrometer (Edinburgh Instruments Ltd., Livingston, United Kingdom). Absolute PL quantum yield was measured *via* an integrating light sphere. Solutions were placed in 1 cm path length quartz cells. CD spectra were recorded using Chirascan-Plus CD (Applied Photophysics, United Kingdom) spectrometers in 1 cm path length quartz cells. CPL spectra were recorded using JASCO CPL-300 spectrofluorescence polarimeter (Tokyo, Japan) in 1 cm path length quartz cells.

Single-crystal X-ray diffractions. X-ray diffraction data for **(R)-S1** were collected using synchrotron radiation and MAR325 CCD detector at Shanghai Synchrotron Radiation BL17B Beamline. Indexing was performed using APEX3.[1] Data integration and reduction was performed using SaintPlus.[2] Absorption correction was performed by multi-scan method implemented in SADABS.[3] Space group was determined using XPREP implemented in APEX3.[1] Structure was solved using SHELXT[4] and was refined using SHELXL-2018[5,6] (full-matrix least-square on F^2) through OLEX2 interface program.[7] Some of disorder counterions and solvent molecules were refined with restraints. Ellipsoid plot was done with Platon.[8]

Computational details. All calculations were carried out with the Gaussian 16 software.[9] Density functional theory (DFT) calculations were carried out with the PBE0 functional with the combination of the Grimme's D3BJ version of dispersion correction.[10] The basis set of 6-31G (d) were adopt for the geometry optimization and frequency calculations. The geometries were fully optimized without any structural constraints. The implicit solvation model SMD with acetonitrile as the solvent was

applied in the calculations. The harmonic frequency calculations were carried out at the same level of theory to verify that all structures have no imaginary frequency. The absorption spectra were calculated by TD-DFT calculation with considering 30 states. The excited states S_1 and S_2 of complex **S1** were optimized with TD-DFT method. The frontier orbitals were rendered by Vesta program.[11] The electric magnetic dipole moments were analyzed by Multiwfn program[12] and visualized with the VMD program[13].

2. Synthesis and characterization of ligands and complexes

2.1 Synthetic routes of ligands

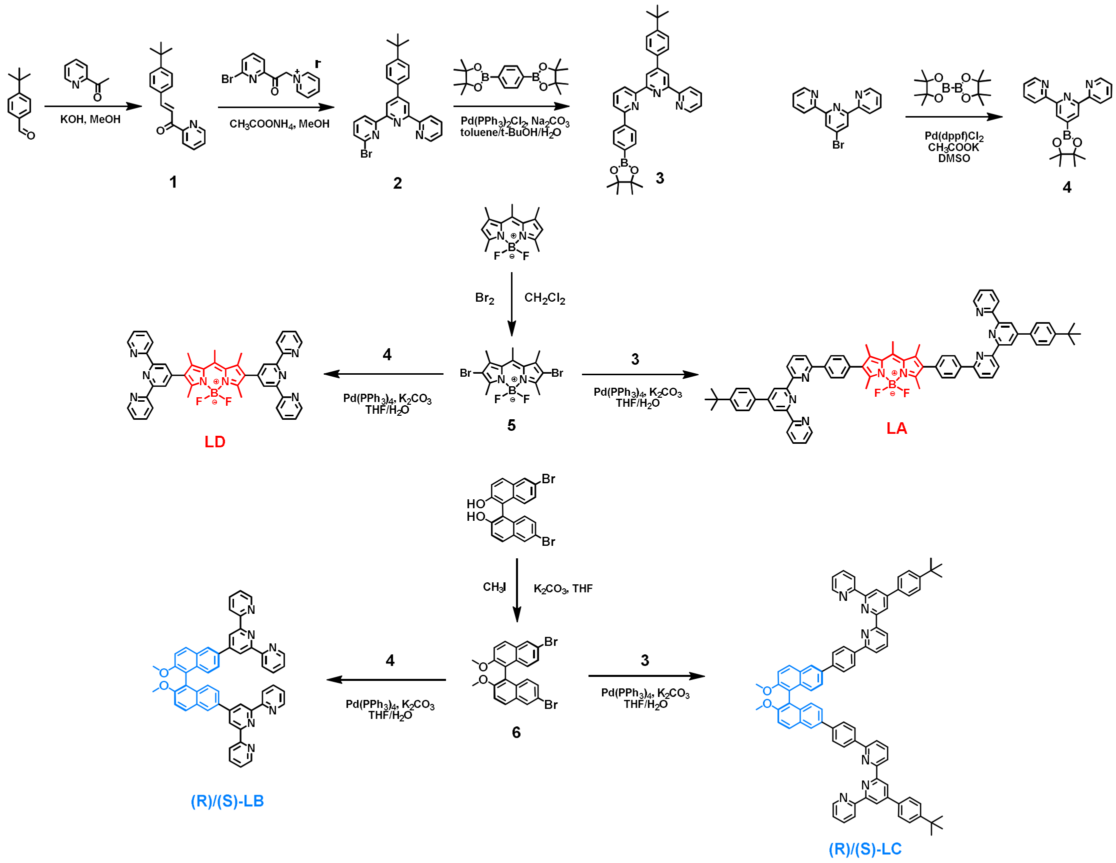

Figure S1. Synthesis of ligands **LA**, **LB**, **LC**, and **LD**.

2.2 Synthesis of ligands and complexes

Compound **3**[14], compound **4**[15], compound **5**[16], compound **6**[14] and ligand **LC**[14] were prepared according to previous reports. The ligand **LB**[17] has been documented in the literature.

Ligand LA

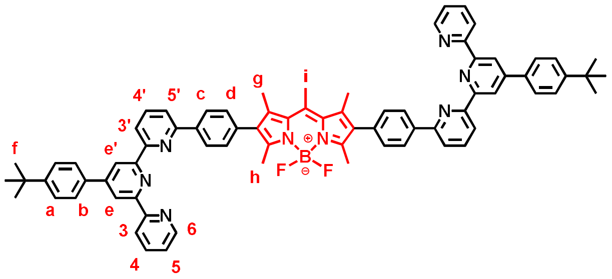

A mixture of compound **3** (0.9 mmol, 510.8 mg), compound **5** (0.4 mmol, 168.0 mg), and $Pd(PPh_3)_4$ (0.07 mmol, 80.0 mg) in 100 mL Schlenk flask was degassed three times. Then 32.0 mL THF and 10.0 mL 1 M K_2CO_3 were added under N_2. The resultant mixture was kept at 65 ºC for 48 h. After cooling down to the room temperature, the solution was extracted three times with $CHCl_3$, and the solvent was removed under reduced pressure. The crude product was purified by column chromatography on silica gel ($CHCl_3$/EtOH = 100/1) to give ligand **LA** as red solid (205.2 mg, 45%). ^{1}H NMR (400 MHz, $CDCl_3$) δ 8.95 (d, J = 1.7 Hz, 2H, tpy-$H^{e'}$), 8.77 – 8.69 (m, 6H, tpy-H^3, tpy-H^6, and tpy-H^e), 8.67 (dd, J = 7.8, 0.9 Hz, 2H, tpy-$H^{3'}$), 8.28 (d, J = 8.3 Hz, 4H, Ph-H^c), 7.98 (t, J = 7.8 Hz, 2H, tpy-$H^{4'}$), 7.88 (m, 8H, Ph-H^b, tpy-$H^{5'}$, and tpy-H^4), 7.60 – 7.56 (m, 4H, Ph-H^a), 7.42 (d, J = 8.2 Hz, 4H, Ph-H^d), 7.39 – 7.34 (m, 2H, tpy-H^5), 2.76 (s, 3H, H^i), 2.60 (s, 6H, H^g), 2.44 (s, 6H, H^h), 1.41 (s, 18H, H^f). ^{13}C NMR (100 MHz, $CDCl_3$) δ 156.27, 156.22, 156.16, 156.12, 155.50, 152.62, 152.43, 150.37, 148.98, 137.83, 137.36, 135.91, 133.36, 130.97, 127.24, 127.17, 126.10, 123.97, 121.66, 120.48, 119.93, 119.21, 119.20, 34.90, 31.48, 17.50, 15.78, 13.57. MALDI-TOF MS (*m/z*): Calcd. for $[C_{76}H_{67}BF_2N_8+H]^+$ 1141.56. Found: 1141.56.

Ligand LD

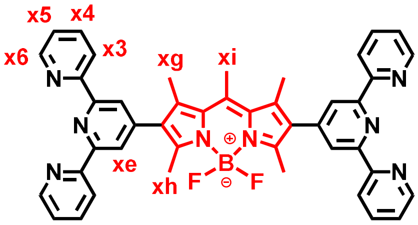

A mixture of compound **4** (1.2 mmol, 431.0 mg), compound **5** (0.5 mmol, 210.0 mg), and $Pd(PPh_3)_4$ (0.07 mmol, 80.0 mg) in 100 mL Schlenk flask was degassed three times. Then 24.0 mL THF and 6.0 mL 1 M K_2CO_3 were added under N_2. The resultant mixture was kept at 65 ºC for 48 h. After cooling to the room temperature, the solution was extracted by $CHCl_3$ three times, and the solvent was removed under reduced pressure. The crude product was purified by column chromatography on silica gel ($CHCl_3$/EtOH = 50/1) to give ligand **LD** as brown solid (130.0 mg, 36%). ^{1}H NMR (400 MHz, $CDCl_3$) δ 8.73 – 8.66 (m, 8H, tpy-H^{x6} and tpy-H^{x3}), 8.38 (s, 4H, tpy-H^{xe}), 7.89 (td, J = 7.7, 1.8 Hz, 4H, tpy-H^{x4}), 7.35 (ddd, J = 7.5, 4.7, 1.2 Hz, 4H, tpy-H^{x5}), 2.78 (s, 3H, H^{xi}), 2.62 (s, 6H, H^{xg}), 2.48 (s, 6H, H^{xh}). ^{13}C NMR (100 MHz, $CDCl_3$) δ 156.18, 155.77, 152.70, 149.36, 144.28, 142.88, 137.99, 137.06, 132.71, 131.81, 124.03, 122.64, 121.45, 17.58, 15.95, 13.63. MALDI-TOF MS (*m/z*): Calcd. for $[C_{44}H_{35}BF_2N_8+H]^+$ 725.30. Found: 725.30.

Ligand LB

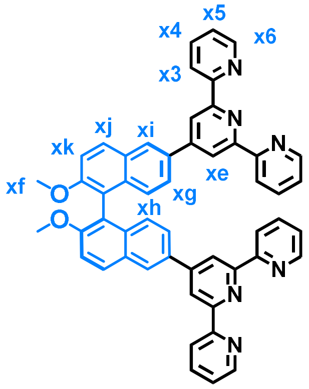

A mixture of compound **4** (0.5 mmol, 179.6 mg), compound **6** (0.2 mmol, 94.0 mg), K_2CO_3 (1.4 mmol, 193.2 mg) and $Pd(PPh_3)_4$ (0.03 mmol, 40.0 mg) in 100 mL Schlenk flask was degassed three times. Then 20.0 mL THF and 6 mL H_2O were added under N_2. The resultant mixture was kept at 65 ºC for 48 h. After cooling down to the room temperature, the solution was extracted three times with $CHCl_3$, and the solvent was removed under reduced pressure. The crude product was purified by column chromatography on silica gel ($CHCl_3$/EtOH = 75/1) to give ligand **LB** as white solid (104.0 mg, 67%). ^{1}H NMR (400 MHz, $CDCl_3$) δ 8.85 (s, 2H, tpy-H^{xe}), 8.73 (ddd, J = 4.8, 1.8, 0.9 Hz, 2H, tpy-H^{x6}), 8.68 (dt, J = 8.0, 1.1 Hz, 2H, tpy-H^{x3}), 8.48 (d, J = 1.9 Hz, 1H, H^{xi}), 8.16 (d, J = 9.0 Hz, 1H, H^{xh}), 7.88 (td, J = 7.7, 1.8 Hz, 2H, tpy-H^{x4}), 7.81 (dd, J = 8.9, 1.9 Hz, 1H, H^{xj}), 7.56 (d, J = 9.1 Hz, 1H, H^{xg}), 7.35 (ddd, J = 7.5, 4.8, 1.2 Hz, 2H, tpy-H^{x5}), 7.30 (d, J = 8.9 Hz, 1H, H^{xk}), 3.85 (s, 3H, H^{xf}). ^{13}C NMR (100 MHz, $CDCl_3$) δ 156.48, 156.01, 155.82, 150.38, 149.24, 136.94, 134.31, 133.50, 130.51, 129.36, 127.04, 126.17, 125.63, 123.87, 121.49, 119.23, 118.97, 114.70, 56.92. MALDI-TOF MS (m/z): Calcd. for $[C_{52}H_{36}N_6O_2+H]^+$ 777.29. Found: 777.29.

Complex S1

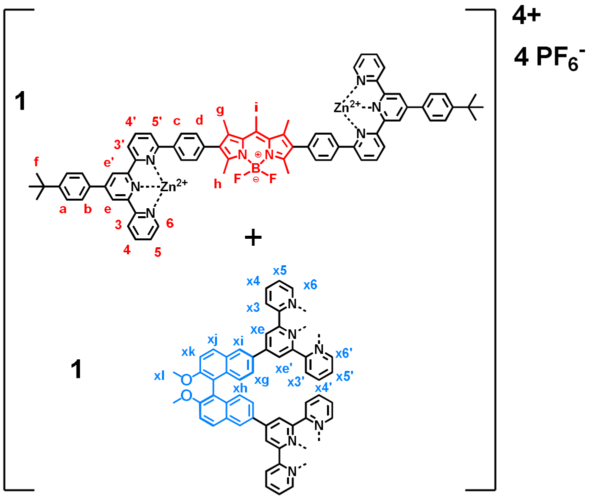

To a solution of ligand **LA** (26.0 μmol, 29.7 mg) and ligand **LB** (26.0 μmol, 20.2 mg) in $CHCl_3$ (50.0 mL), a solution of $Zn(NO_3)_2\cdot 6H_2O$ (52.0 μmol, 15.5 mg) in MeOH (100.0 mL) was added, then the mixture was kept in 50 ºC for 10 h. After cooling to room temperature, excess NH_4PF_6 (around 1.0 g) was added to generate a yellow precipitate (62.1 mg, 91%). ^{1}H NMR (600 MHz, CD_3CN) δ 9.13 (s, 2H, tpy-$H^{xe'}$), 9.04 (s, 2H, tpy-$H^{e'}$), 8.93 (s, 2H, tpy-H^{xe}), 8.89 – 8.83 (m, 4H, tpy-H^{e} and tpy-$H^{x3'}$), 8.73 – 8.69 (m, 4H, H^{xi} and tpy-$H^{3'}$), 8.61 – 8.55 (m, 4H, tpy-H^{3} and tpy-H^{x3}), 8.41 – 8.34 (m, 4H, H^{xj} and tpy-$H^{x4'}$), 8.28 (td, J = 7.7, 1.7 Hz, 2H, tpy-$H^{x4'}$), 8.23 – 8.18 (m, 6H, H^{b} and tpy-H^{x4}), 8.04 – 7.97 (m, 4H, tpy-H^{4} and H^{xg}), 7.93 – 7.90 (m, 2H, tpy-H^{x6}), 7.86 (d, J = 4.9 Hz, 2H, tpy-$H^{x6'}$), 7.84 – 7.80 (m, 6H, H^{a} and H^{xk}), 7.55 – 7.48 (m, 6H, tpy-$H^{x5'}$, tpy-H^{x5} and tpy-$H^{5'}$), 7.45 (d, J = 8.8 Hz, 2H, H^{xh}), 7.23 (dd, J = 7.1, 5.8 Hz, 2H, tpy-H^{5}), 6.93 (d, J = 6.3 Hz, 2H, tpy-H^{6}), 6.78 (d, J = 8.0 Hz, 4H, H^{d}), 6.59 (d, J = 8.0 Hz, 4H, H^{c}), 3.84 (s, 6H, H^{xl}), 2.41 (s, 3H, H^{i}), 2.17 (s, 6H, H^{g}), 2.10 (s, 6H, H^{h}), 1.47 (s, 18H, H^{f}). ^{13}C NMR (125 MHz, CD_3CN) δ 161.75, 157.76, 157.01, 156.11, 156.04, 152.45, 152.16, 151.16, 150.72, 149.36, 149.29, 149.25, 149.15, 149.00, 148.89, 148.63, 148.47, 147.74, 144.11, 142.50, 142.45, 142.35, 141.63, 138.64, 137.44, 135.87, 135.61, 134.02, 132.99, 132.41, 132.27, 131.20, 130.58, 130.21, 129.91, 129.69, 128.86, 128.63, 128.46, 128.41, 127.68, 127.02, 125.49, 124.23, 124.16, 123.92, 123.76, 122.61, 122.31, 121.53, 121.43, 119.54, 116.31, 57.16, 35.71, 31.39, 17.97, 15.86, 13.88. ESI-MS (m/z): 511.2 $[M-4PF_6^-]^{4+}$ (calcd m/z: 511.2), 729.9 $[M-3PF_6^-]^{3+}$ (calcd m/z: 729.9), 1167.3 $[M-2PF_6^-]^{2+}$ (calcd m/z: 1167.3). Elemental analysis for **S1** ($C_{128}H_{103}BF_2N_{18}O_{14}Zn_2$, NO_3^- as anions): C 65.98% (calcd: 66.93%), H 4.61% (calcd: 4.52%), N 10.08% (calcd: 10.98%), S 0.00% (calcd: 0.00%).

Complex S2

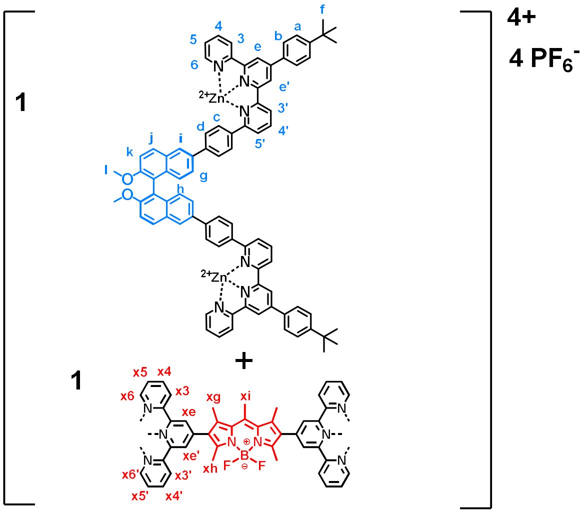

To a solution of ligand **LC** (26.0 μmol, 31.0 mg) and ligand **LD** (26.0 μmol, 18.8 mg) in $CHCl_3$ (50.0 mL), a solution of $Zn(NO_3)_2 \cdot 6H_2O$ (52.0 μmol, 15.5 mg) in MeOH (100.0 mL) was added, then the mixture was kept in 50 ºC for 10 h. After cooling to room temperature, excess NH_4PF_6 (around 1.0 g) was added to generate a red precipitate (63.5 mg, 93%). ^{1}H NMR (600 MHz, CD_3CN) δ 9.13 (s, 2H, tpy-$H^{e'}$), 8.97 (s, 2H, tpy-H^{e}), 8.85 (d, J = 8.1 Hz, 2H, tpy-$H^{3'}$), 8.62 (d, J = 8.1 Hz, 2H, tpy-H^{3}), 8.58 (d, J = 8.0 Hz, 2H, tpy-$H^{x3'}$), 8.50 (d, J = 8.0 Hz, 2H, tpy-H^{x3}), 8.38 – 8.31 (m, 6H, tpy-$H^{4'}$, tpy-H^{xe} and tpy-$H^{xe'}$), 8.27 (t, J = 7.7 Hz, 2H, tpy-$H^{x4'}$), 8.21 (d, J = 8.1 Hz, 6H, H^{b} and tpy-H^{x4}), 8.15 (d, J = 9.0 Hz, 2H, H^{j}), 8.06 (t, J = 7.7 Hz, 2H, tpy-H^{4}), 8.02 (s, 2H, H^{i}), 7.95 (d, J = 4.2 Hz, 2H, tpy-$H^{x6'}$), 7.88 (d, J = 5.2 Hz, 2H, tpy-H^{x6}), 7.84 (d, J = 7.9 Hz, 4H, H^{a}), 7.60 (d, J = 9.0 Hz, 2H, H^{k}), 7.54 (d, J = 7.4 Hz, 4H, tpy-$H^{5'}$ and tpy-$H^{x5'}$), 7.49 (t, J = 6.2 Hz, 2H, tpy-H^{x5}), 7.40 – 7.31 (m, 6H, H^{h}, H^{g} and tpy-H^{6}), 7.30 – 7.23 (m, 6H, H^{d} and tpy-H^{5}), 6.66 (d, J = 7.6 Hz, 4H, H^{c}), 3.66 (s, 6H, H^{l}), 2.68 (s, 3H, H^{xi}), 2.55 (s, 6H, H^{xg}), 2.50 (s, 6H, H^{xh}), 1.48 (s, 18H, H^{f}). ^{13}C NMR (125 MHz, CD_3CN) δ 161.75, 157.06, 156.49, 156.14, 156.03, 154.12, 152.24, 151.15, 150.60, 150.01, 149.18, 149.08, 148.99, 148.75, 148.49, 148.36, 148.30, 148.23, 146.42, 142.57, 142.48, 142.40, 142.06, 141.77, 140.79, 137.60, 134.61, 134.12, 134.07, 133.76, 131.45, 130.51, 130.27, 129.97, 129.64, 129.06, 128.87, 128.70, 128.34, 127.69, 127.46, 127.35, 125.49, 124.78, 124.72, 124.35, 124.29, 123.93, 123.79, 122.70, 122.30, 119.17, 115.48, 56.82, 35.72, 31.40, 18.56, 16.47, 14.32. ESI-MS (*m*/*z*): 511.2 $[M\text{-}4PF_6^-]^{4+}$ (calcd *m*/*z*: 511.2), 729.9 $[M\text{-}3PF_6^-]^{3+}$ (calcd *m*/*z*: 729.9), 1167.3 $[M\text{-}2PF_6^-]^{2+}$ (calcd *m*/*z*: 1167.3). Elemental analysis for **S2** ($C_{128}H_{103}BF_2N_{18}O_{14}Zn_2$, NO_3^- as anions): C 66.08% (calcd: 66.93%), H 4.58% (calcd: 4.52%), N 10.51% (calcd: 10.98%), S 0.00% (calcd: 0.00%).

2.3 Isotope patterns of complexes

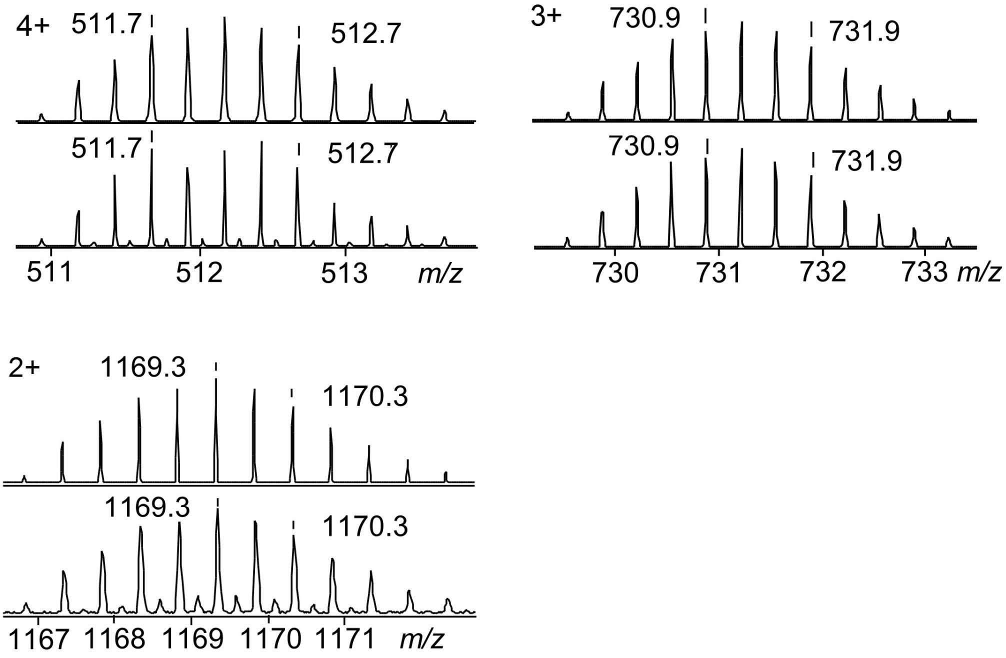

Figure S2. Measured (bottom) and calculated (top) isotope patterns for different charge states observed from complex **S1** (PF_6^- as counterion).

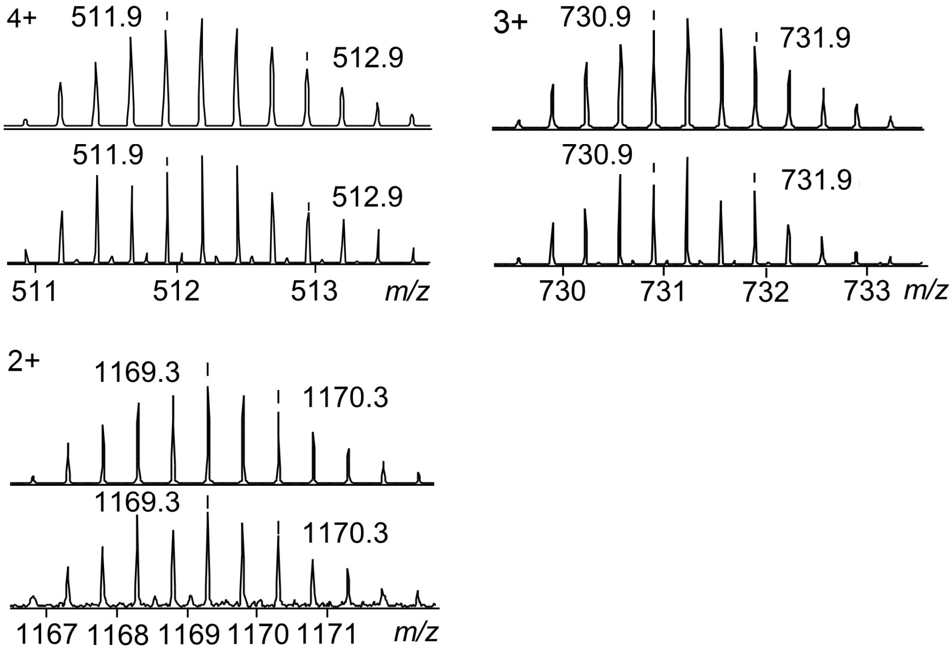

Figure S3. Measured (bottom) and calculated (top) isotope patterns for different states observed from complex **S2** (PF_6^- as counterion).

2.4 ^{1}H NMR, ^{13}C NMR, 2D COSY NMR, 2D NOESY NMR, DOSY NMR and MALDI-TOF

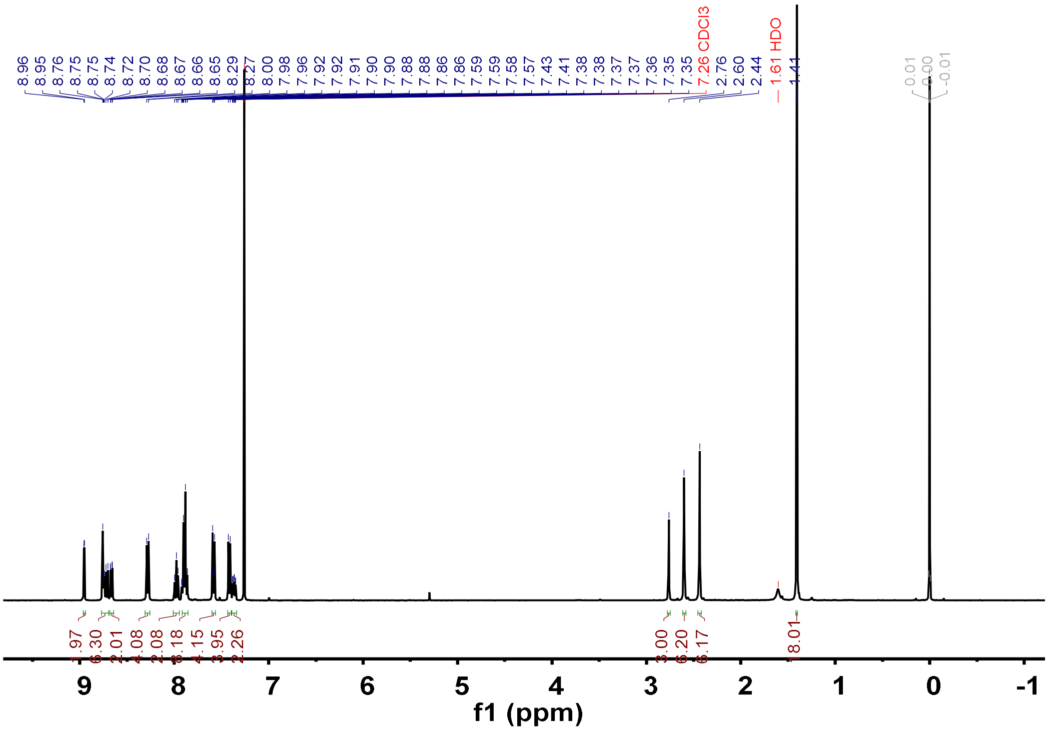

Figure S4. ^{1}H NMR (400 MHz, $CDCl_3$, 300 K) spectrum of ligand **LA**.

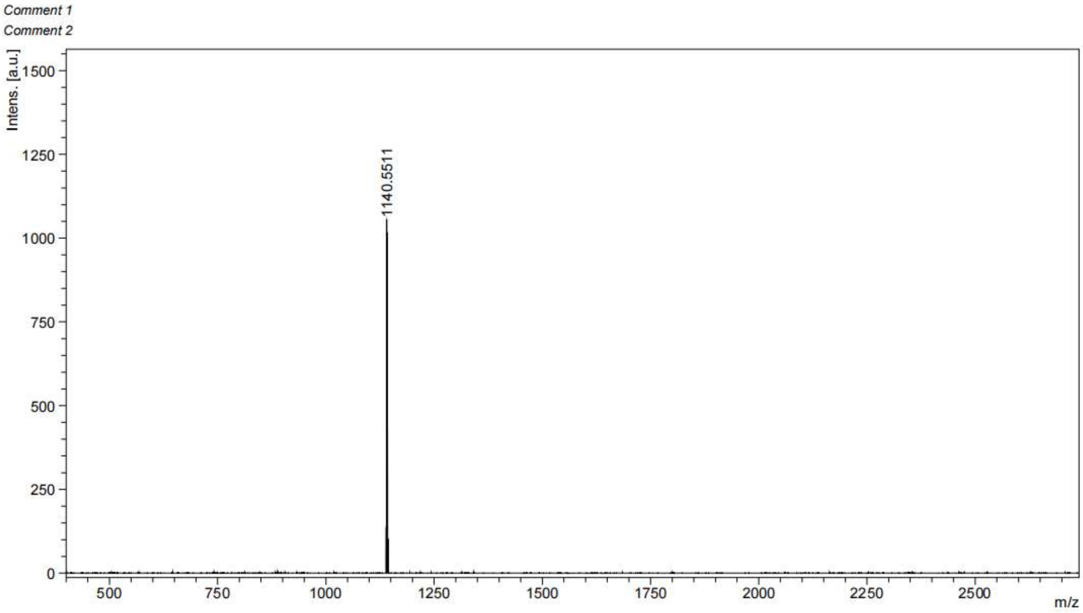

Figure S5. MALDI-TOF plot of ligand **LA**.

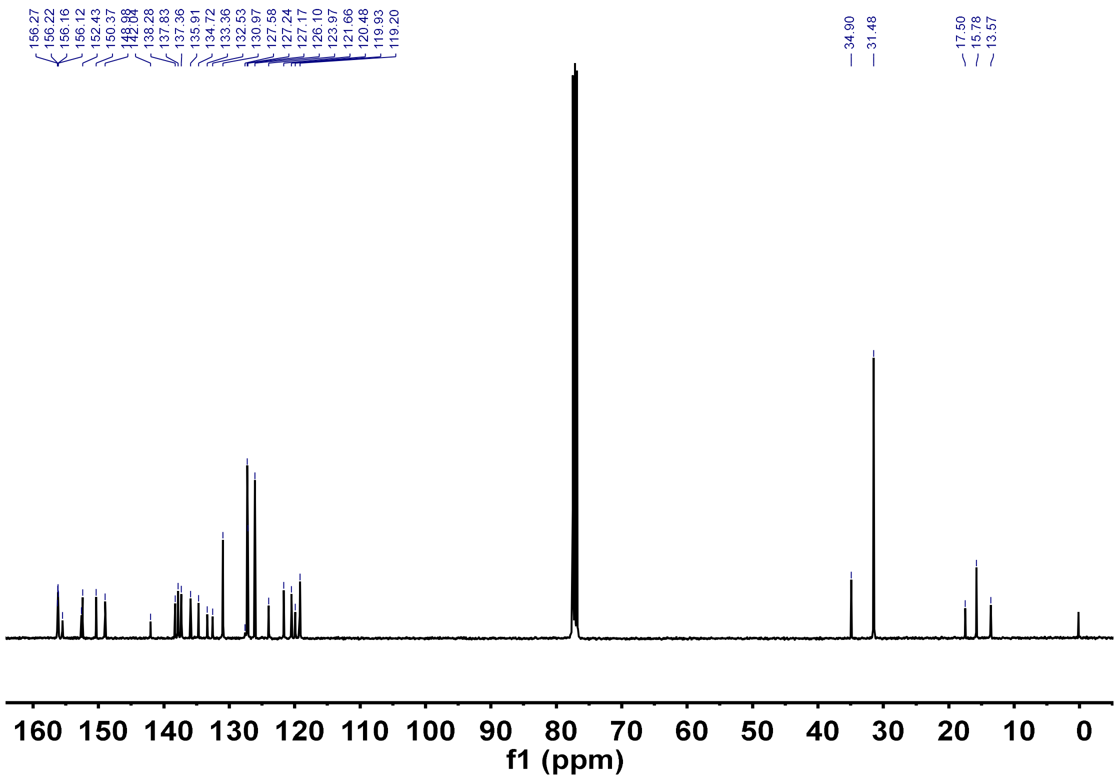

Figure S6. ^{13}C NMR (100 MHz, $CDCl_3$, 300 K) spectrum of ligand **LA**.

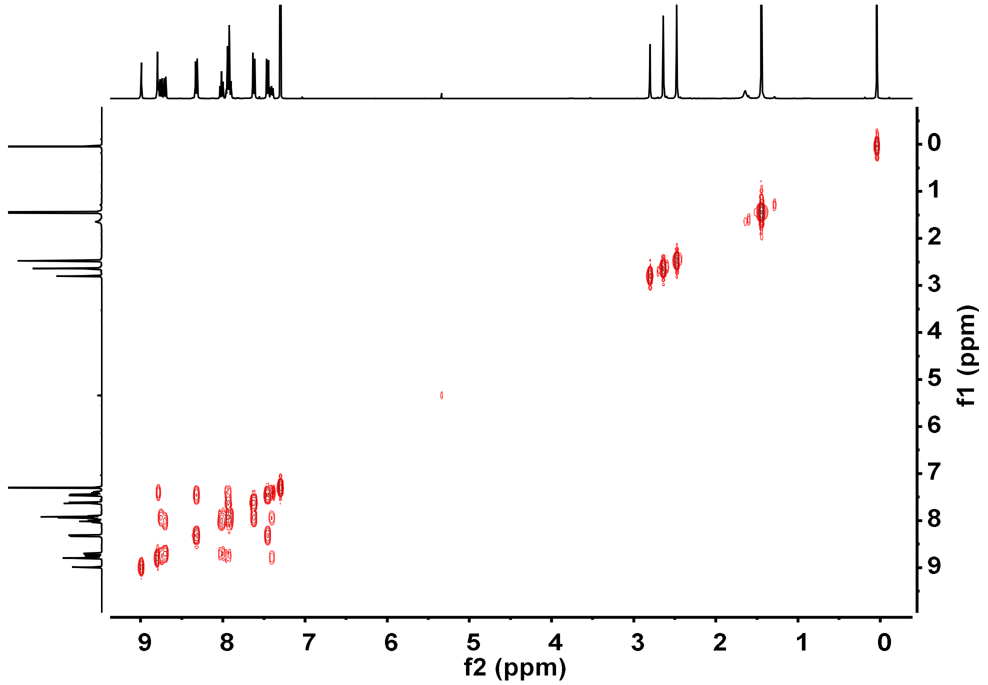

Figure S7. 2D COSY NMR (400 MHz, $CDCl_3$, 300 K) spectrum of ligand **LA**.

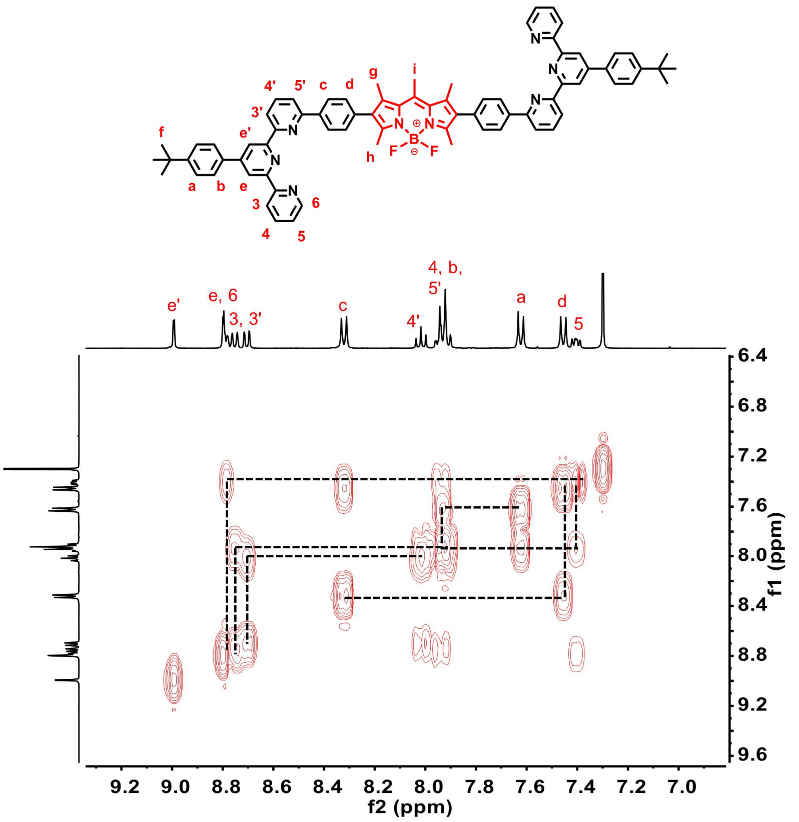

Figure S8. 2D COSY NMR (400 MHz, $CDCl_3$) spectrum of ligand **LA** (aromatic region).

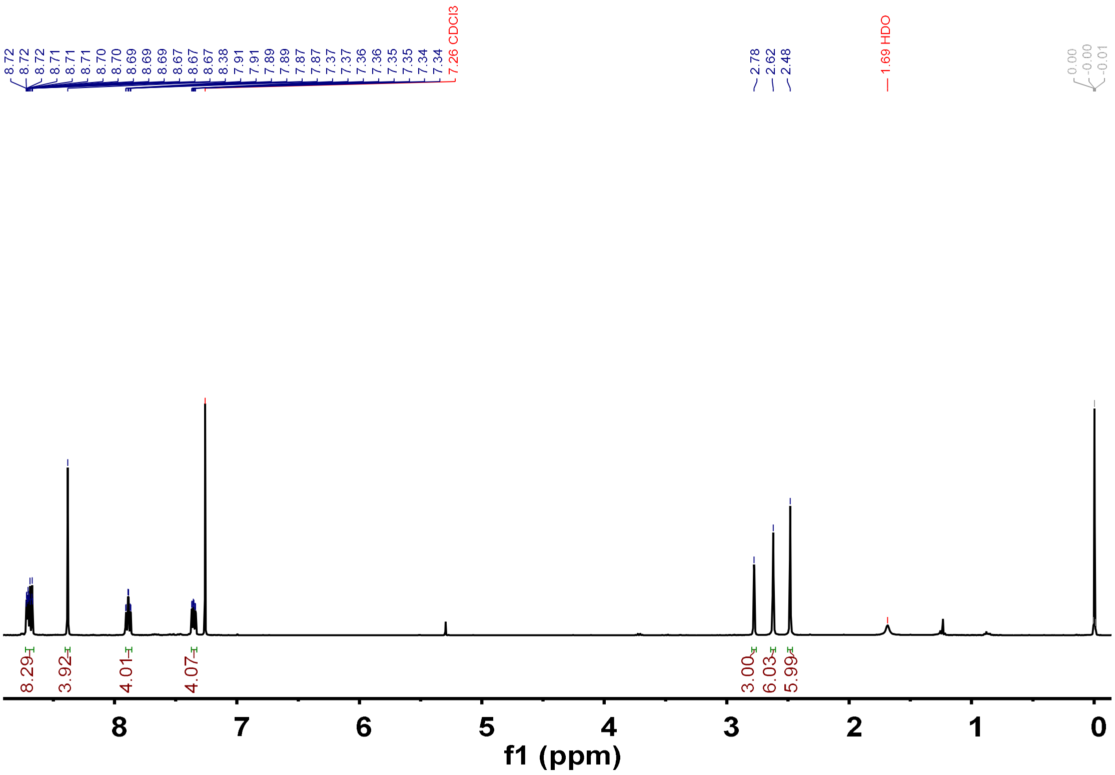

Figure S9. ^{1}H NMR (400 MHz, $CDCl_3$, 300 K) spectrum of ligand **LD**.

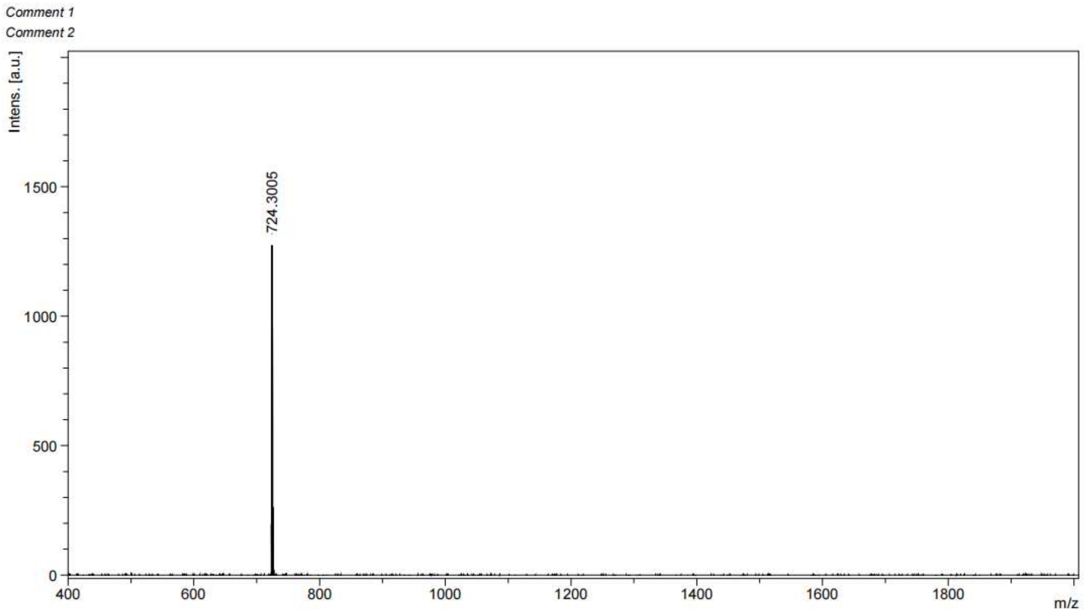

Figure S10. MALDI-TOF plot of ligand **LD**.

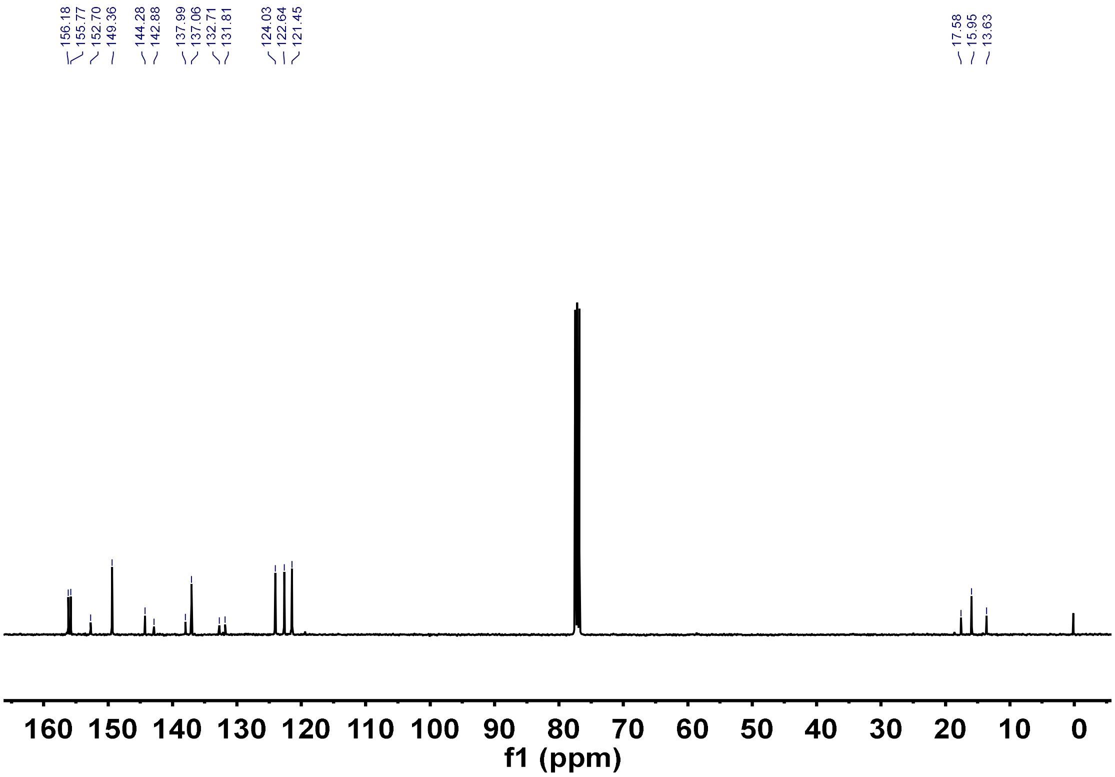

Figure S11. ^{13}C NMR (100 MHz, $CDCl_3$, 300 K) spectrum of ligand **LD**.

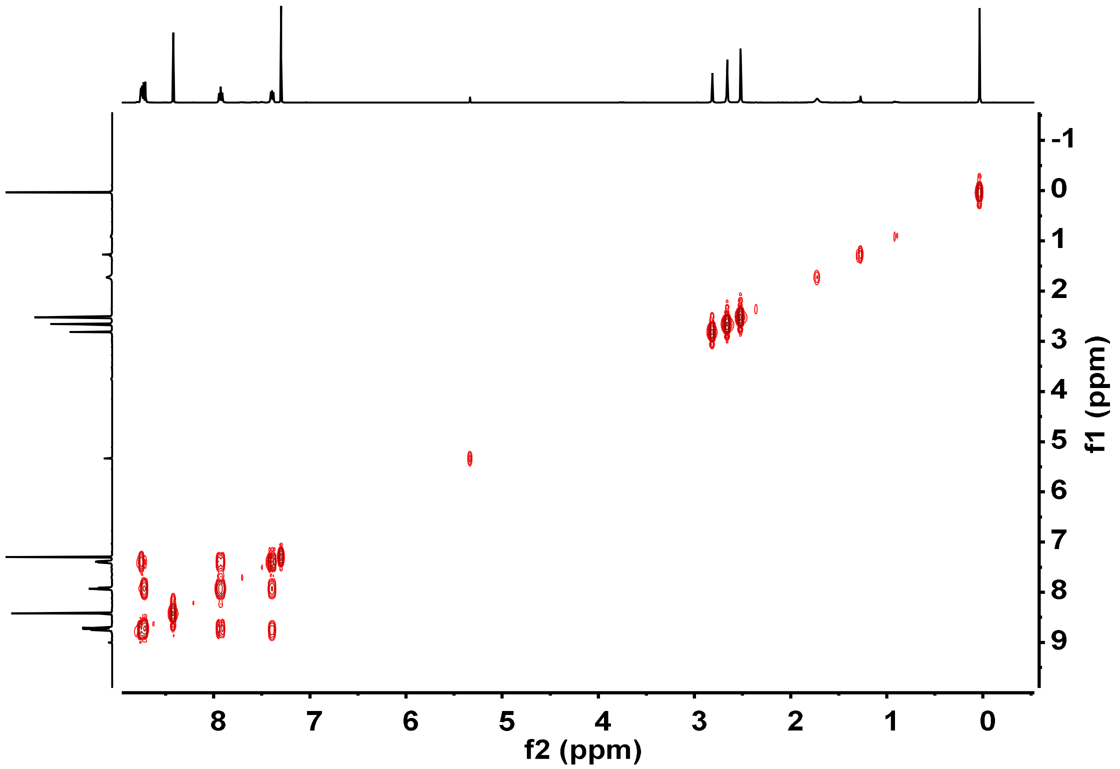

Figure S12. 2D COSY NMR (400 MHz, $CDCl_3$, 300 K) spectrum of ligand **LD**.

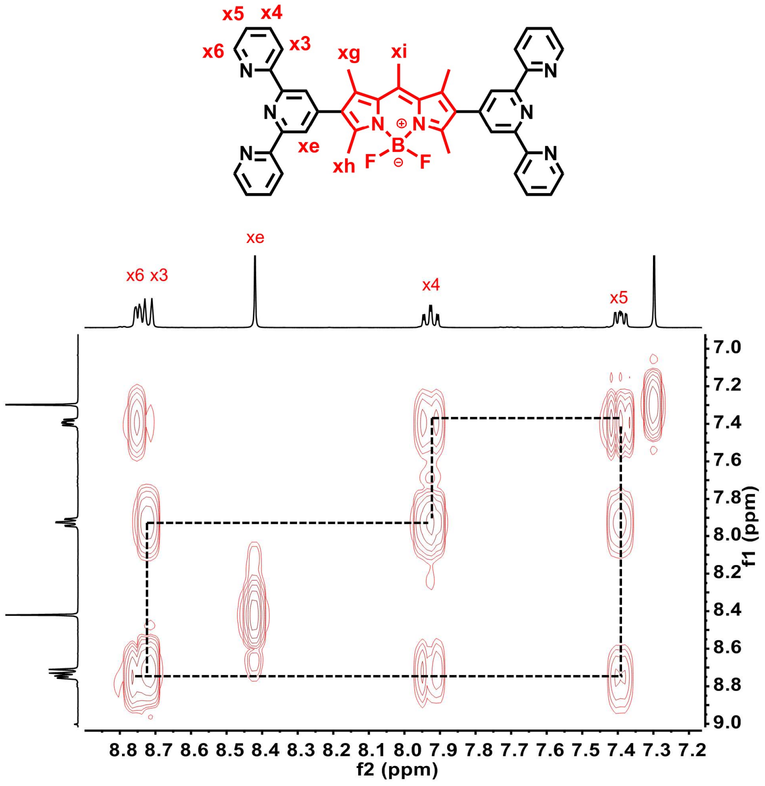

Figure S13. 2D COSY NMR (400 MHz, $CDCl_3$) spectrum of ligand **LD** (aromatic region).

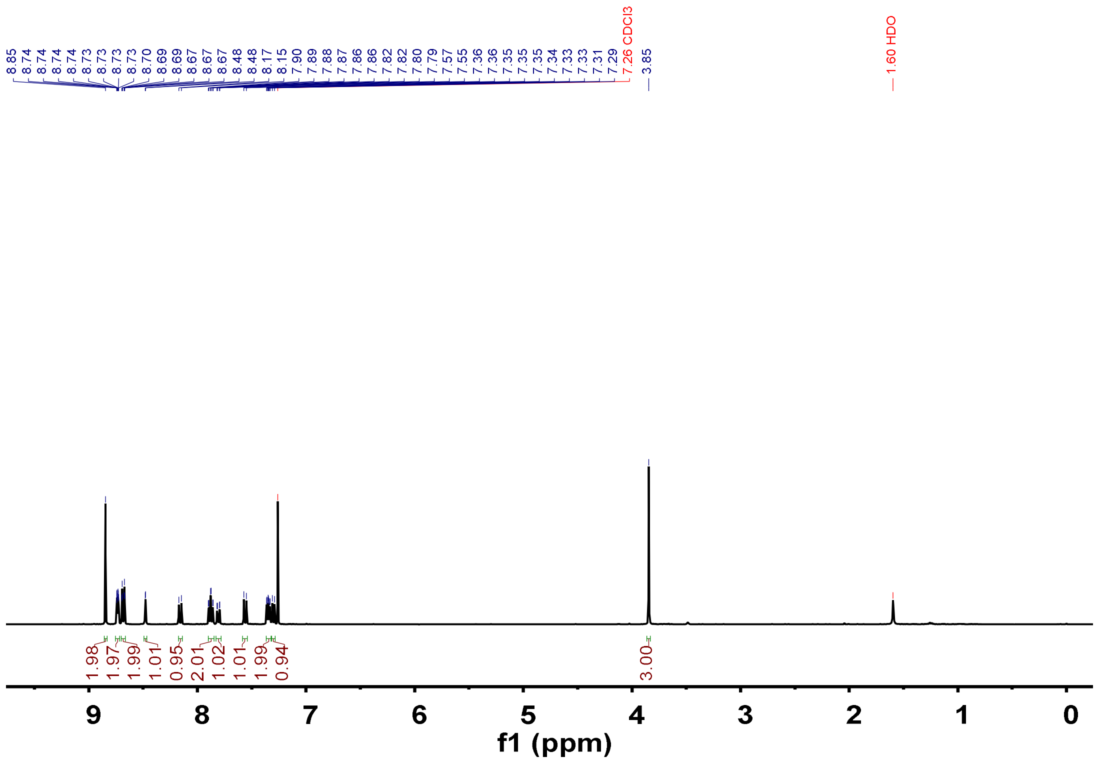

Figure S14. ^{1}H NMR (400 MHz, $CDCl_3$, 300 K) spectrum of ligand **LB**.

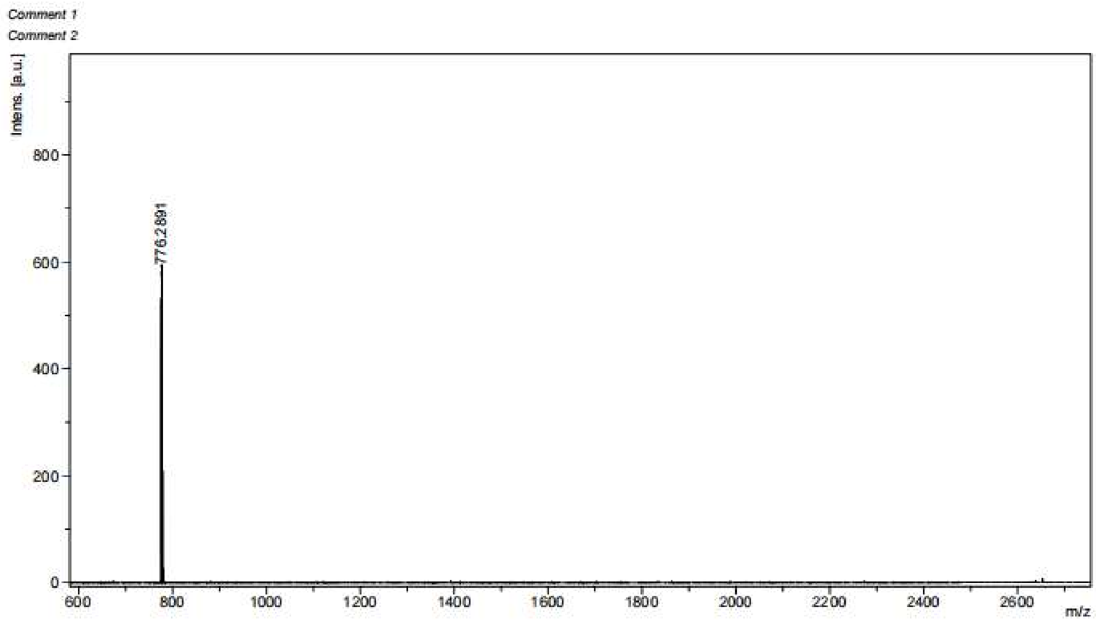

Figure S15. MALDI-TOF plot of ligand **LB**.

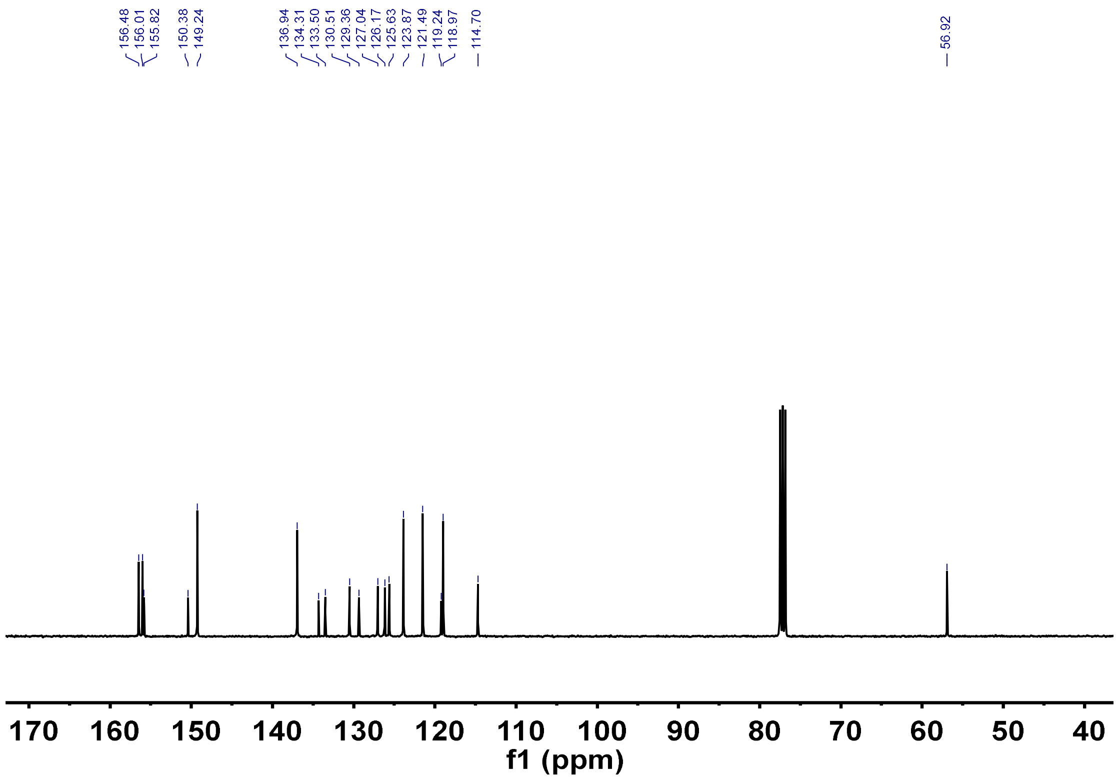

Figure S16. ^{13}C NMR (100 MHz, $CDCl_3$, 300 K) spectrum of ligand **LB**.

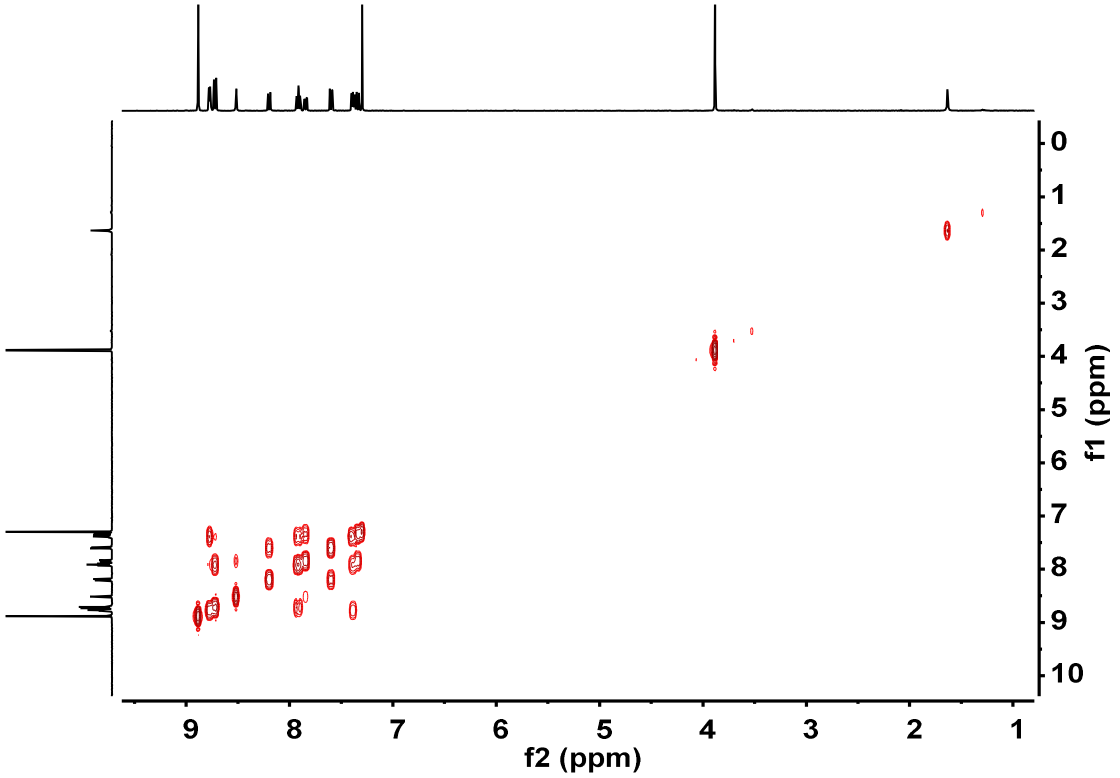

Figure S17. 2D COSY NMR (400 MHz, $CDCl_3$, 300 K) spectrum of ligand **LB**.

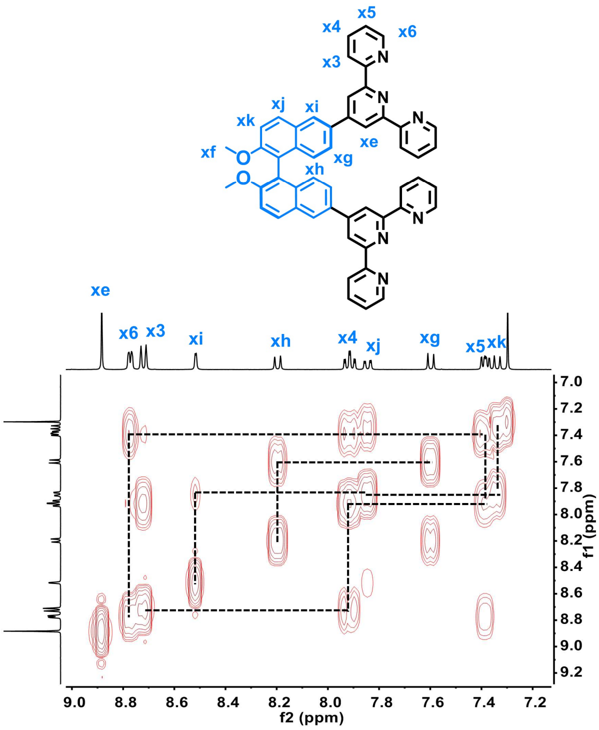

Figure S18. 2D COSY NMR (400 MHz, $CDCl_3$) spectrum of ligand **LB** (aromatic region).

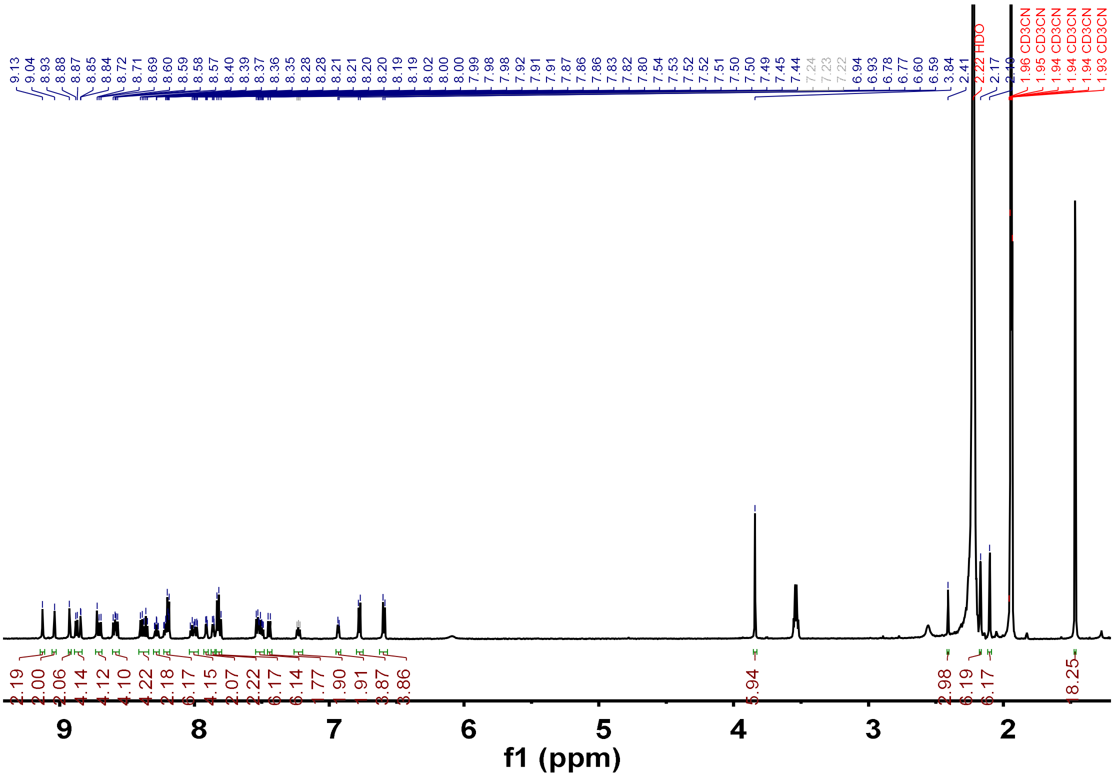

Figure S19. ^{1}H NMR (600 MHz, CD_3CN, 300 K) spectrum of complex **S1**.

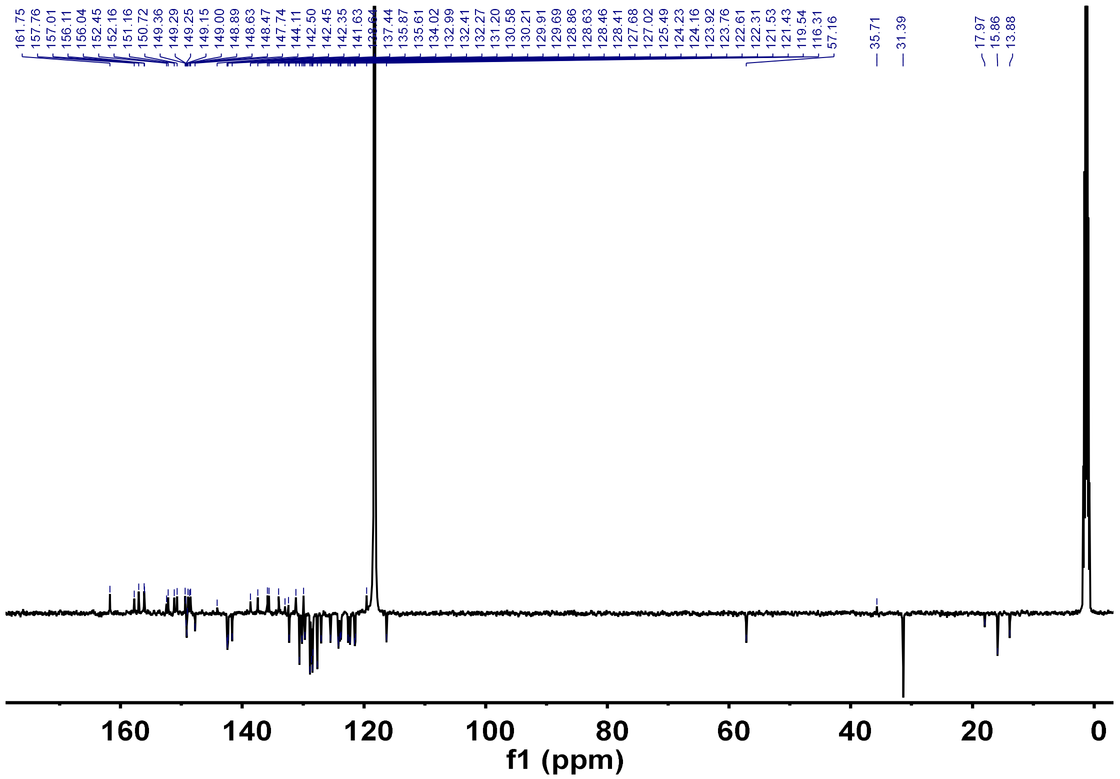

Figure S20. DEPTQ ^{13}C NMR (125 MHz, CD_3CN, 300 K) spectrum of complex **S1**.

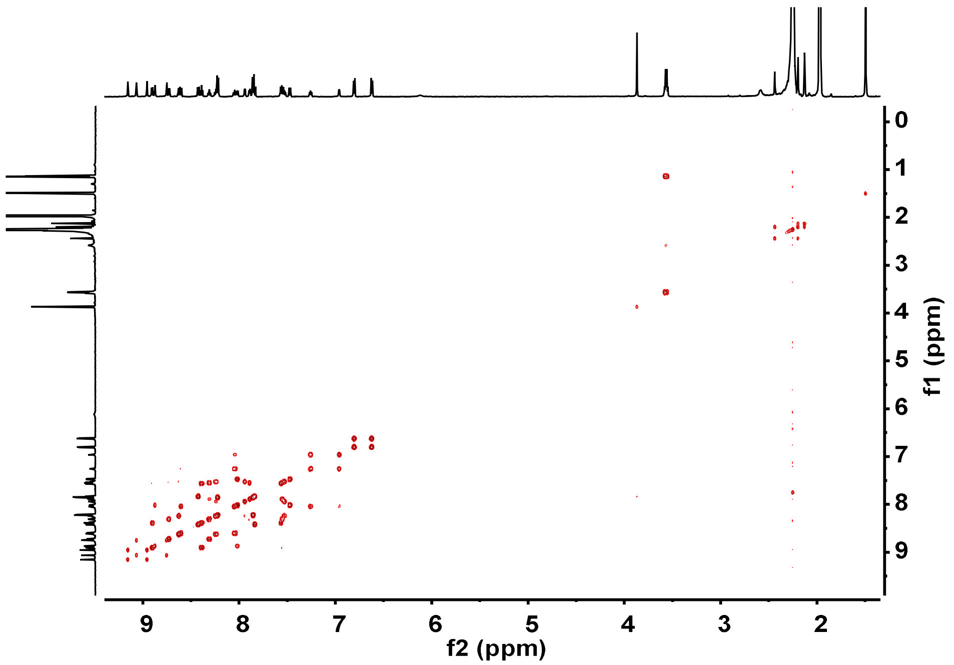

Figure S21. 2D COSY NMR (600 MHz, CD_3CN, 300 K) spectrum of complex **S1**.

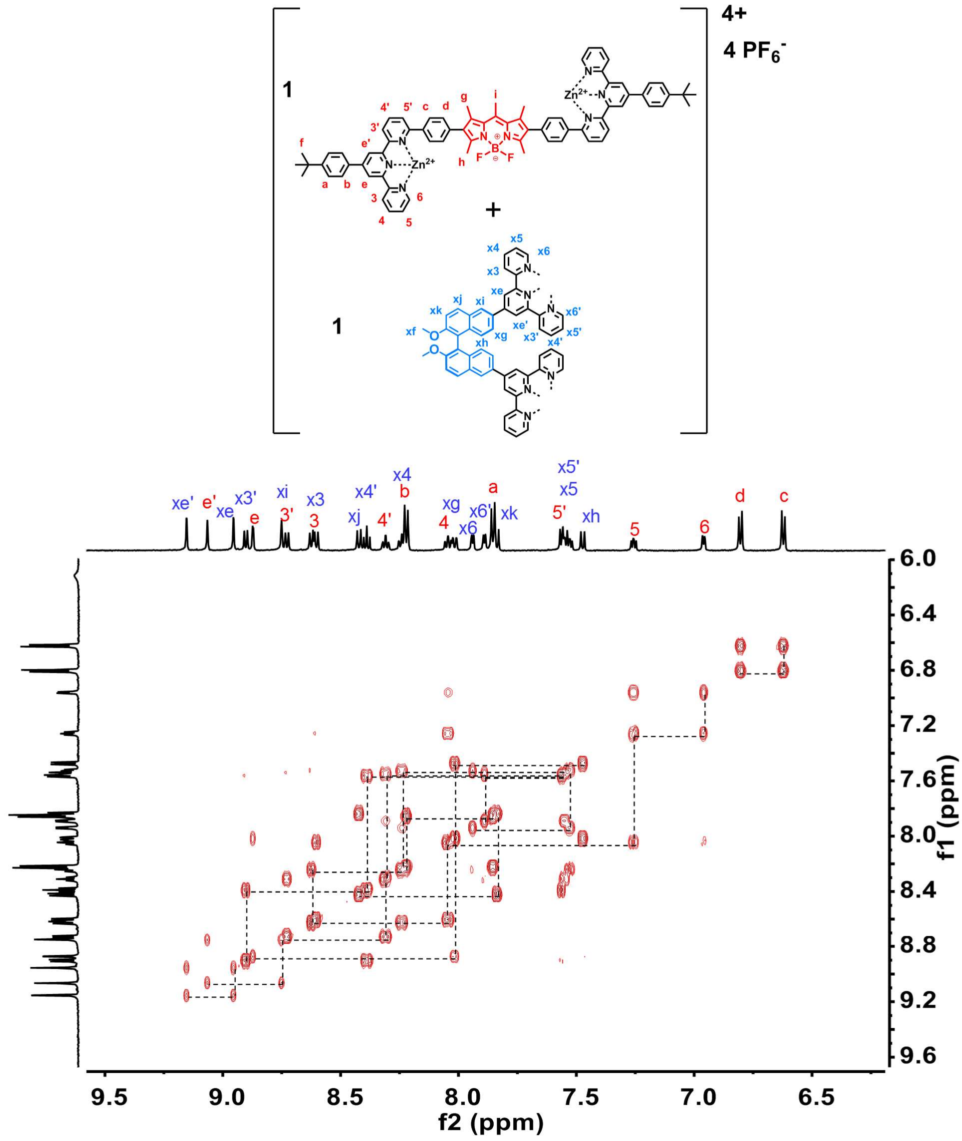

Figure S22. 2D COSY NMR (600 MHz, CD_3CN, 300 K) spectrum of complex **S1** (aromatic region).

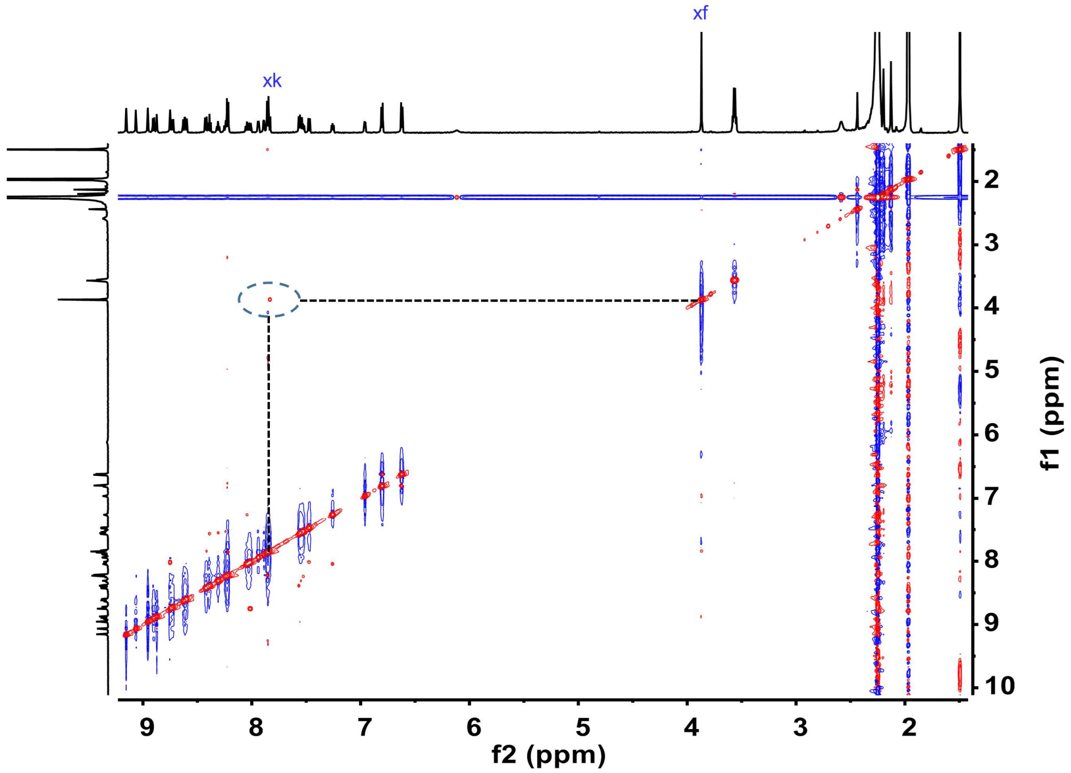

Figure S23. 2D NOESY NMR (600 MHz, CD_3CN, 300 K) spectrum of complex **S1**.

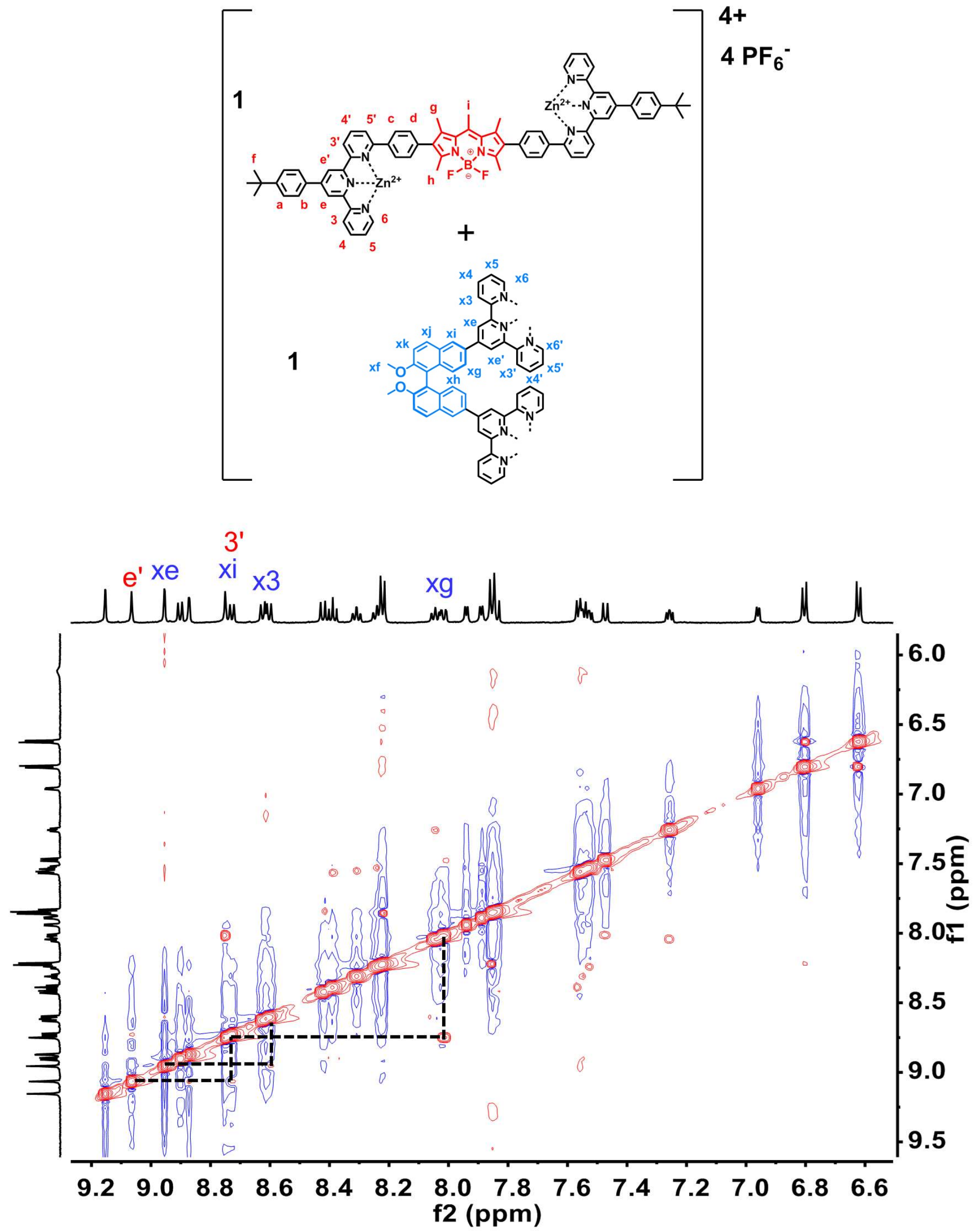

Figure S24. 2D NOESY NMR (600 MHz, CD_3CN, 300 K) spectrum of complex **S1** (aromatic region).

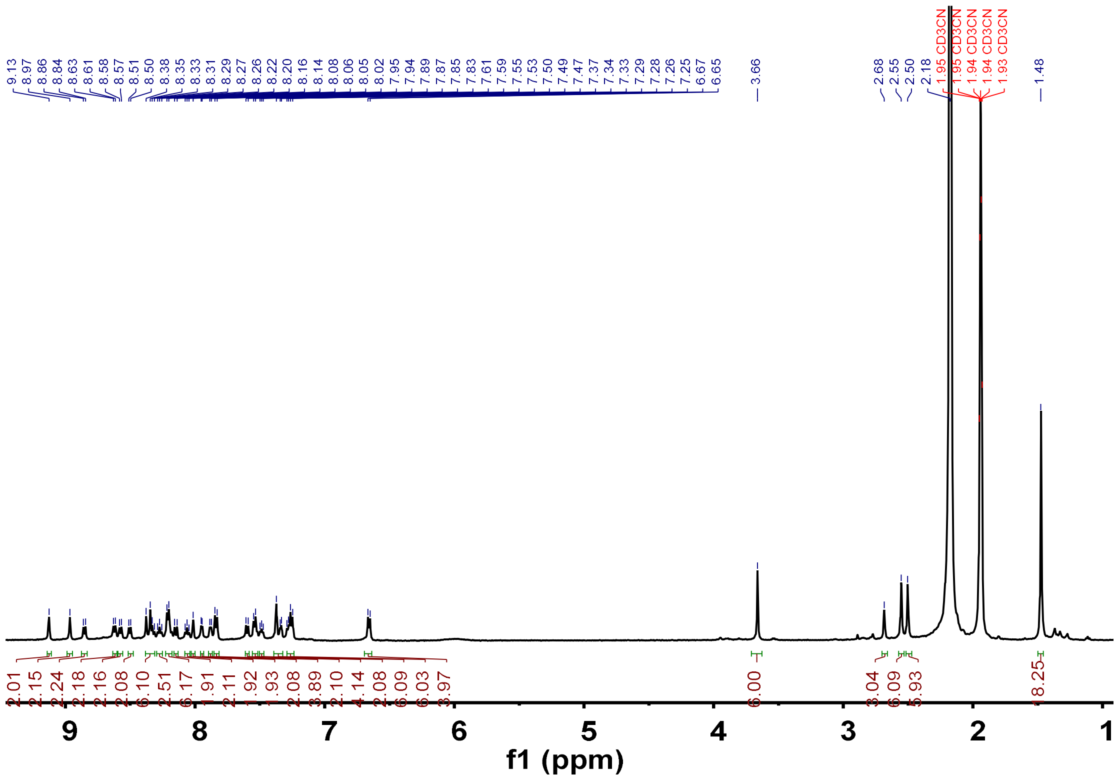

Figure S25. ^{1}H NMR (600 MHz, CD_3CN, 300 K) spectrum of complex **S2**.

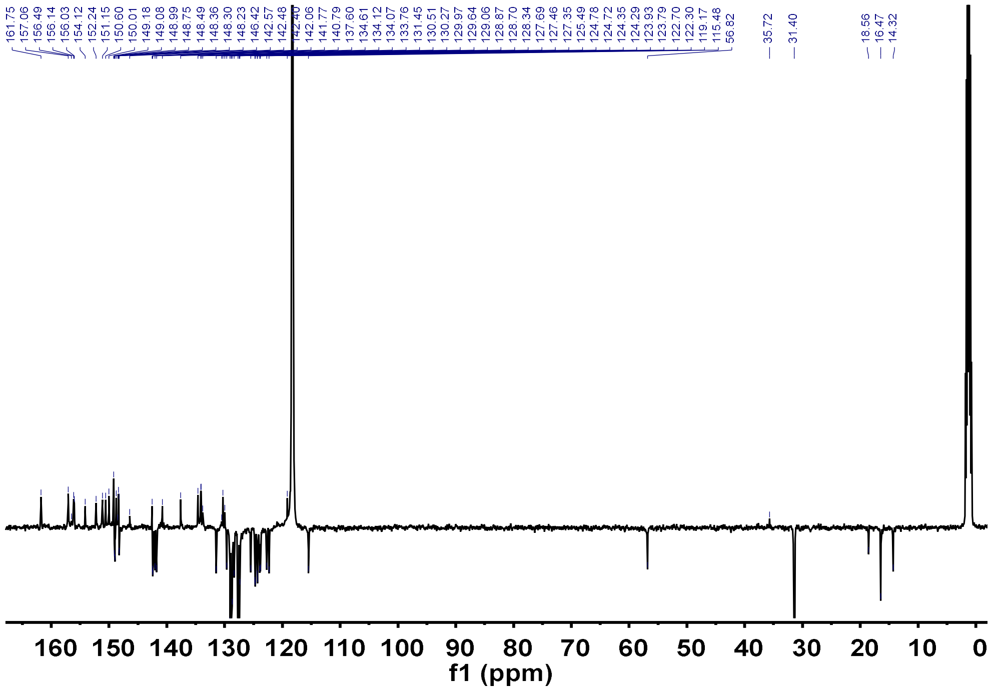

Figure S26. DEPTQ ^{13}C NMR (125 MHz, CD_3CN, 300 K) spectrum of complex **S2**.

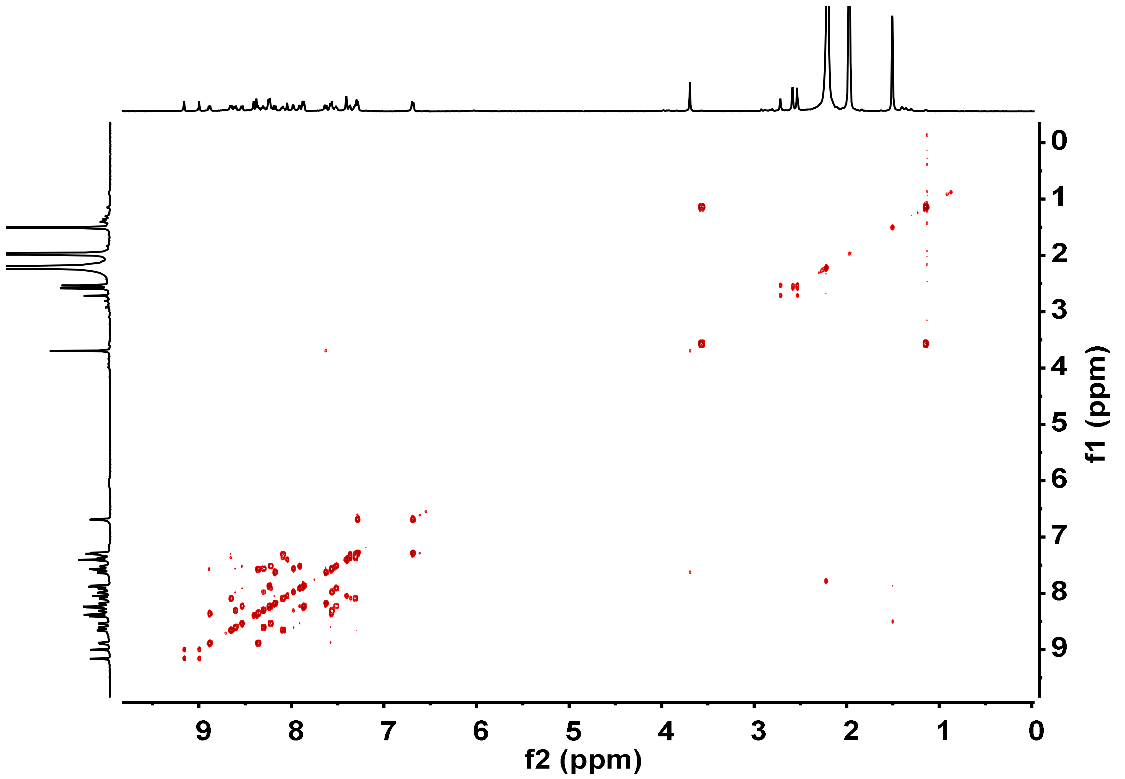

Figure S27. 2D COSY NMR (600 MHz, CD_3CN, 300 K) spectrum of complex **S2**.

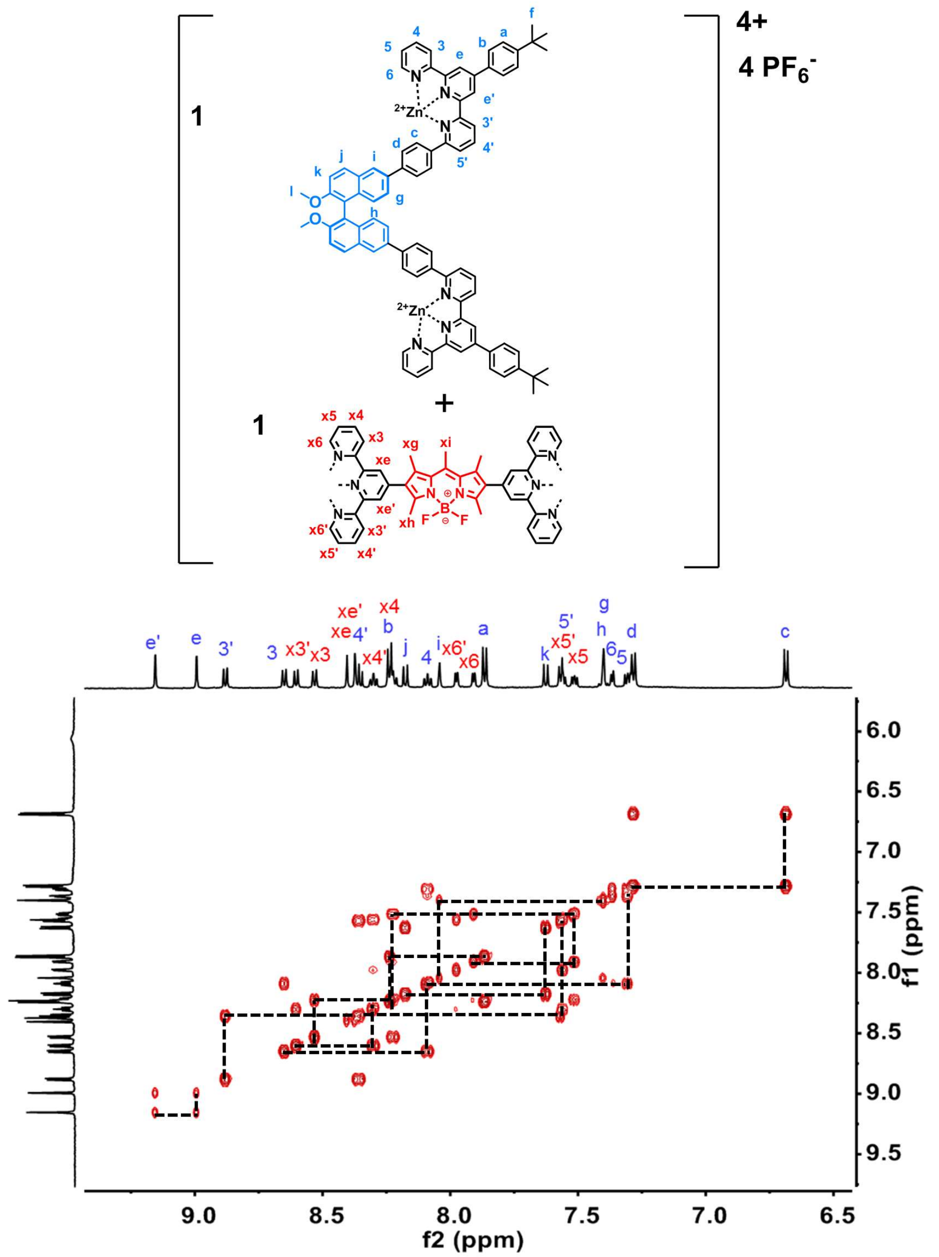

Figure S28. 2D COSY NMR (600 MHz, CD_3CN, 300 K) spectrum of complex **S2** (aromatic region).

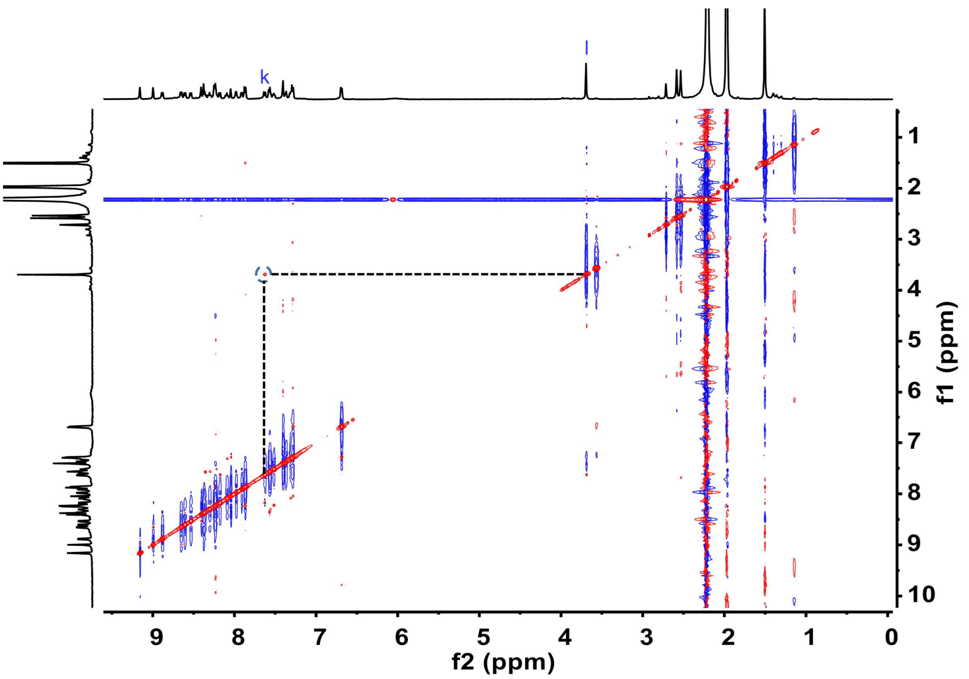

Figure S29. 2D NOESY NMR (600 MHz, CD_3CN, 300 K) spectrum of complex **S2**.

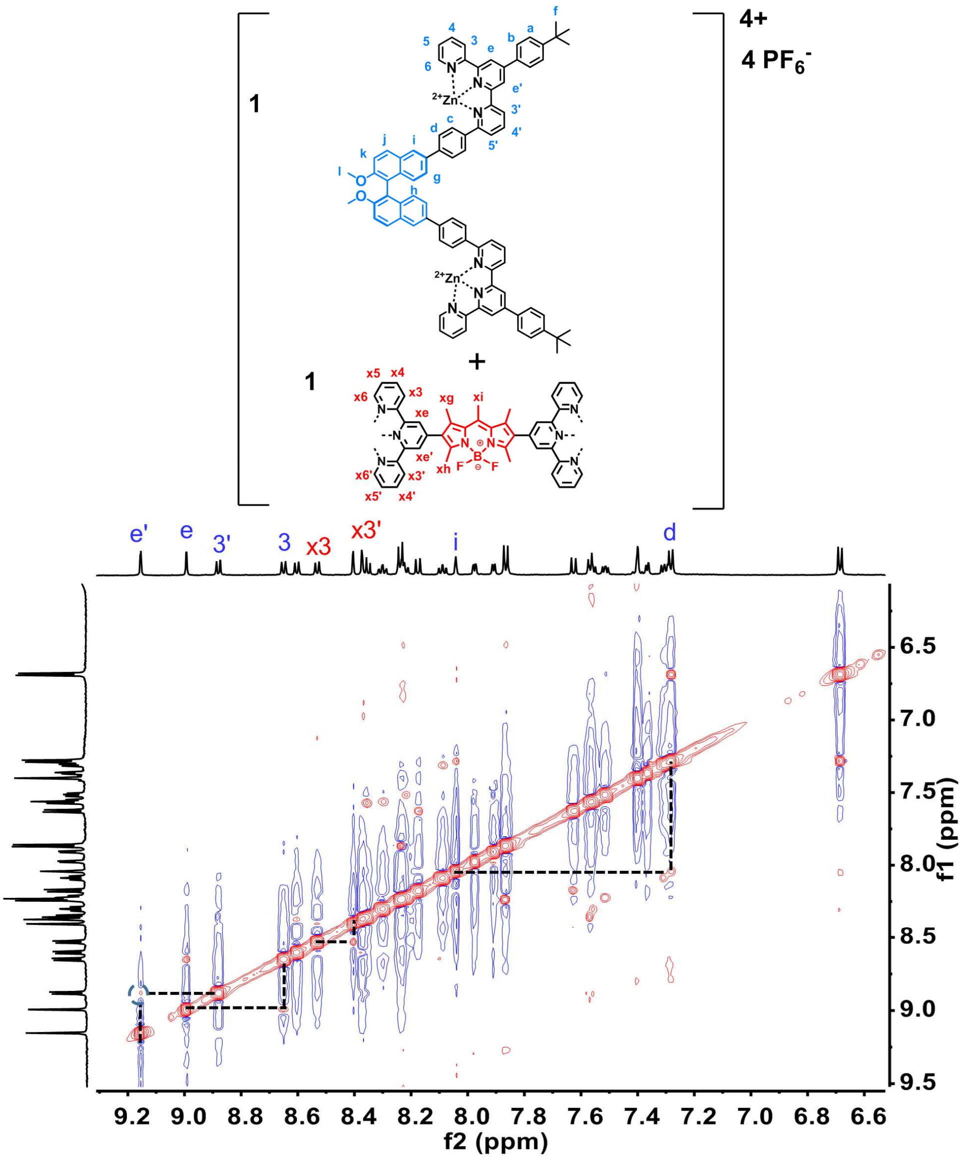

Figure S30. 2D NOESY NMR (600 MHz, CD_3CN, 300 K) spectrum of complex **S2** **(**aromatic region).

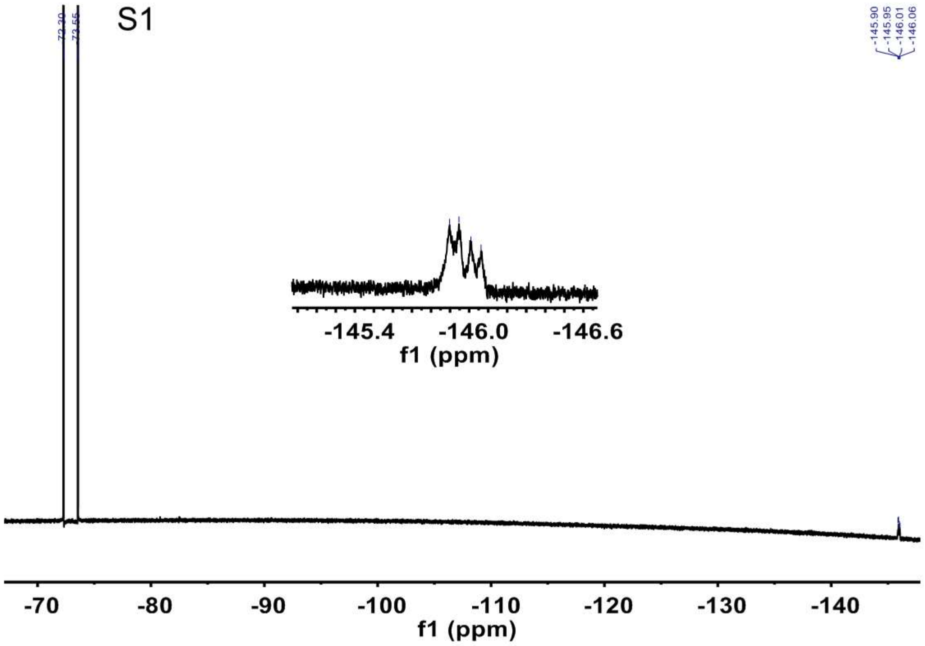

Figure S31. ^{19}F NMR spectrum (500 MHz, 298 K, CD_3CN) of complex **S1**.

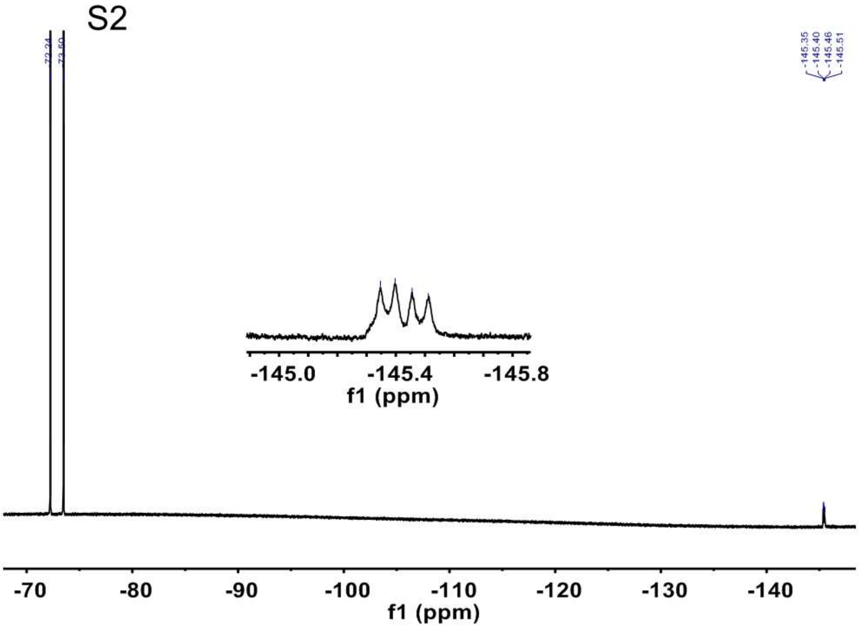

Figure S32. ^{19}F NMR spectrum (500 MHz, 298 K, CD_3CN) of complex **S2**.

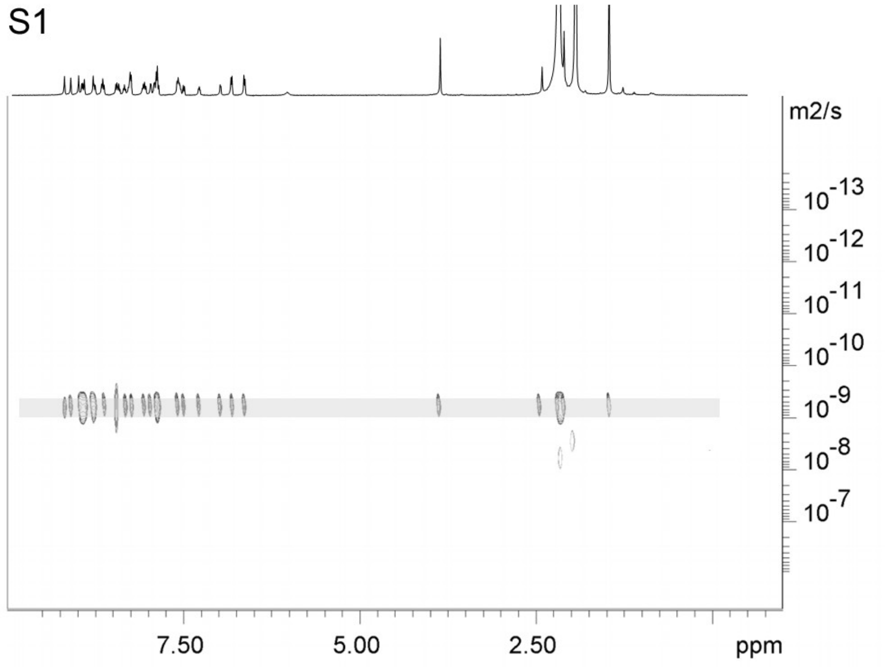

Figure S33. DOSY NMR spectrum (500 MHz, 298 K, CD_3CN) of complex **S1**.

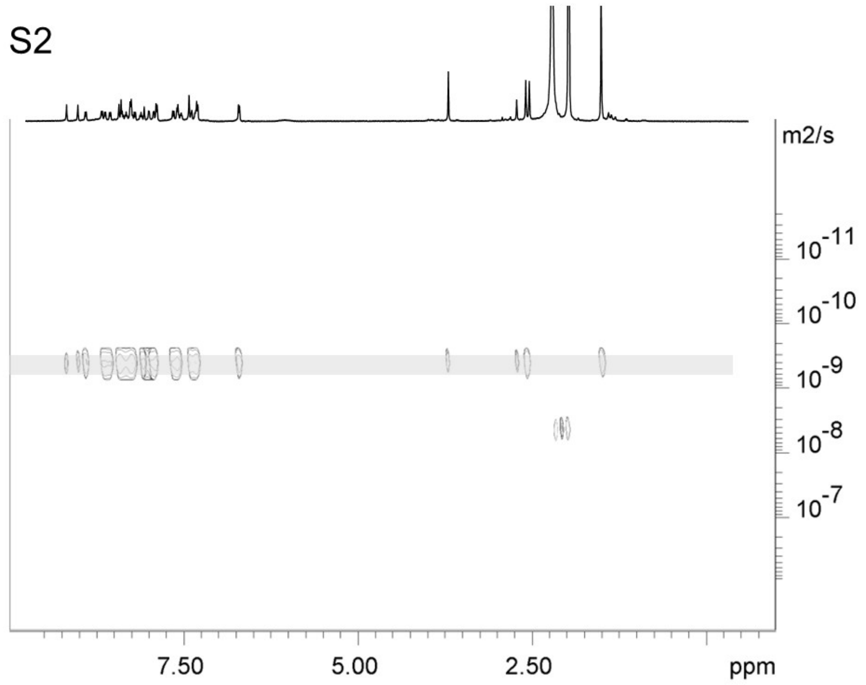

Figure S34. DOSY NMR spectrum (500 MHz, 298 K, CD_3CN) of complex **S2**.

2.5 Photophysical properties of ligands and complexes

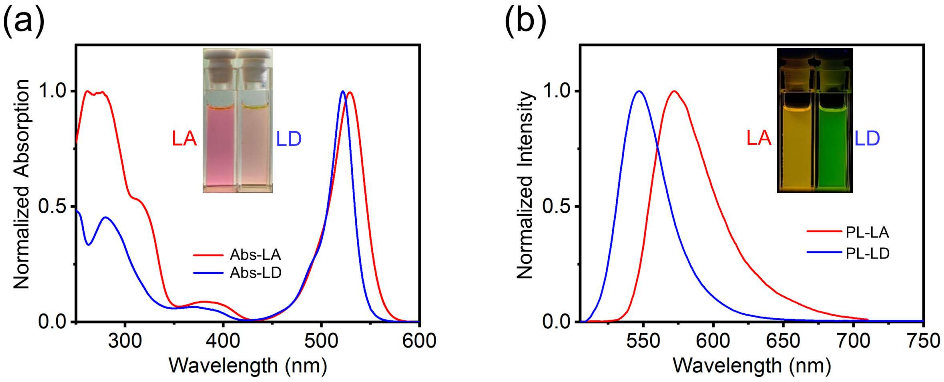

Figure S35. (a) Normalized UV/vis absorption and (b) normalized PL spectra of ligands **LA** and **LD** in $CHCl_3$ (c = 10 μM, λ_{ex} = 515 nm). Insets: photograph of ligands **LA** and **LD** in $CHCl_3$ under visible light (a) and under 365 nm UV light (b).

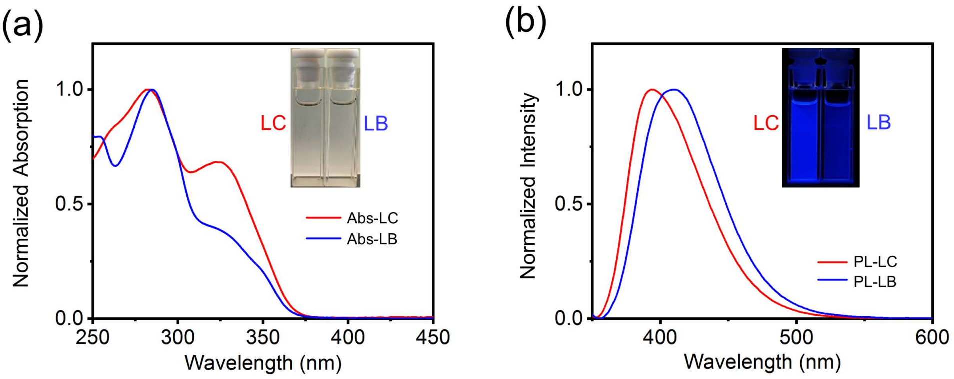

Figure S36. (a) Normalized UV/vis absorption and (b) normalized PL spectra of ligands **LB** and **LC** in $CHCl_3$ (c = 10 μM, λ_{ex} = 330 nm). Insets: photograph of ligands **LB** and **LC** in $CHCl_3$ under visible light (a) and under 365 nm UV light (b).

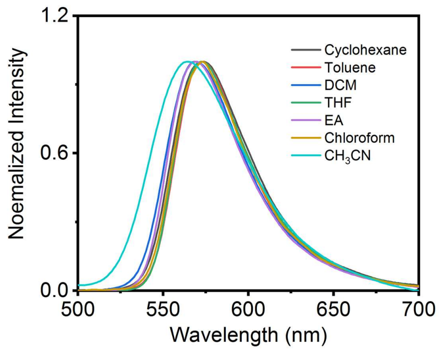

Solvent	λ_{PL}
Cyclohexane	573 nm
Toluene	573 nm
DCM	569 nm
THF	572 nm
EA	569 nm
Chloroform	573 nm
CH_3CN	564 nm

Figure S37. Normalized PL spectra of ligand **LA** in solutions with different polarities. (λ_{ex} = 330 nm).

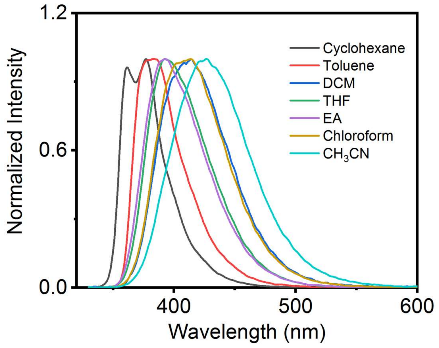

Solvent	λ_{PL}
Cyclohexane	361, 377 nm
Toluene	381 nm
DCM	412 nm
THF	396 nm
EA	395 nm
Chloroform	409 nm
CH_3CN	427 nm

Figure S38. Normalized PL spectra of ligand **LB** in solutions with different polarities. (λ_{ex} = 310 nm).

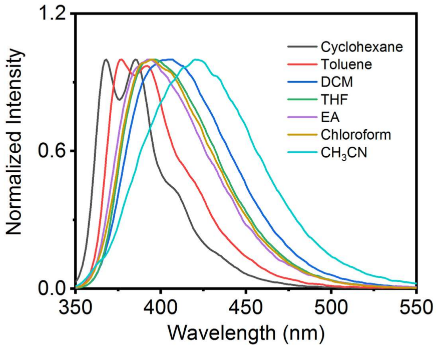

Solvent	λ_{PL}
Cyclohexane	368, 385 nm
Toluene	376, 392 nm
DCM	403 nm
THF	395 nm
EA	394 nm
Chloroform	396 nm
CH_3CN	421 nm

Figure S39. Normalized PL spectra of ligand **LC** in solutions with different polarities. (λ_{ex} = 330 nm).

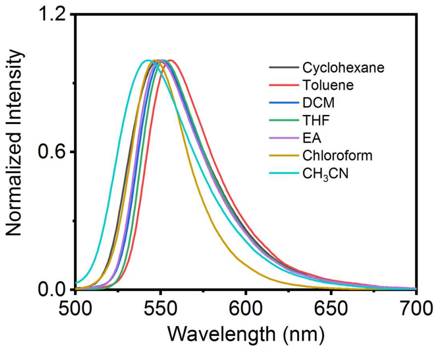

Solvent	λ_{PL}
Cyclohexane	548 nm
Toluene	556 nm
DCM	551 nm
THF	552 nm
EA	550 nm
Chloroform	546 nm
CH_3CN	542 nm

Figure S40. Normalized PL spectra of ligand **LD** in solutions with different polarities. (λ_{ex} = 330 nm).

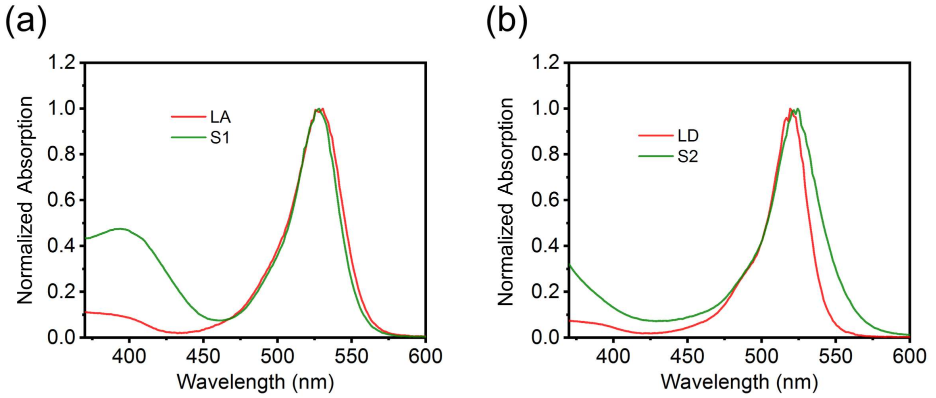

Figure S41. Normalized UV/vis absorption spectra of (a) ligand **LA** and complex **S1** and (b) ligand **LD** and complex **S2** in $CHCl_3$/MeOH (v:v =1:1, c = 10 μM).

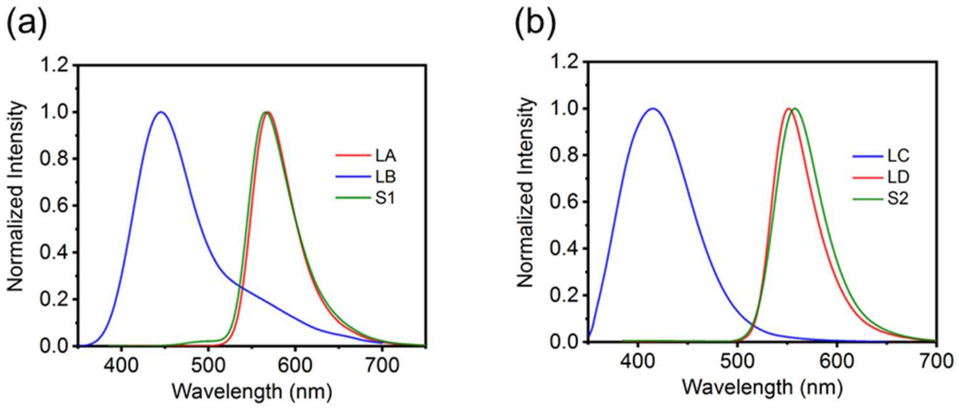

Figure S42. Normalized PL spectra of (a) ligand **LA**, ligand **LB**, and complex **S1** and (b) ligand **LC**, ligand **LD**, and complex **S2** in $CHCl_3$/MeOH (v:v =1:1, c = 10 μM, λ_{ex} = 330 nm).

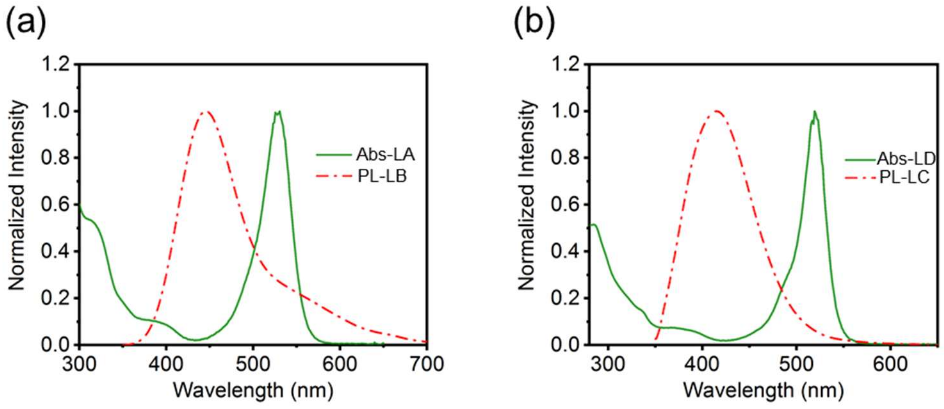

Figure S43. (a) Overlaps of UV/vis absorption spectrum of ligand **LA** and PL spectrum of ligand **LB** in $CHCl_3$/MeOH**.** (b) Overlaps of UV/vis absorption spectrum of ligand **LD** and PL spectrum of ligand **LC** in $CHCl_3$/MeOH. (v:v =1:1, c = 10 μM, λ_{ex} = 330 nm).

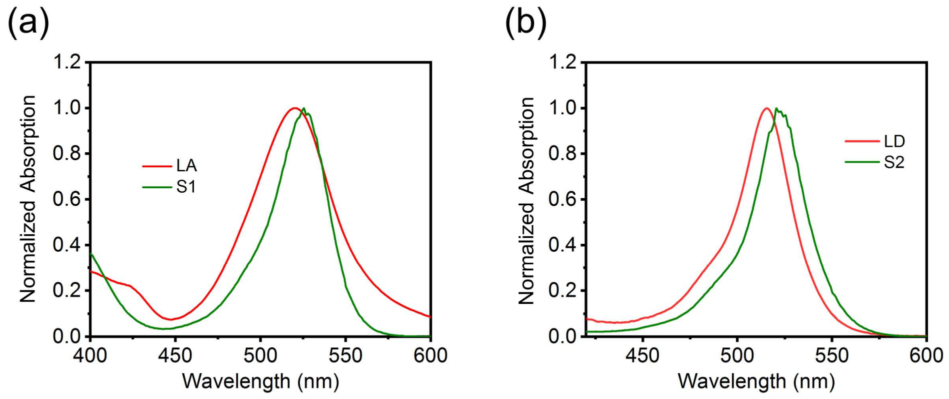

Figure S44. Normalized UV/vis absorption spectra of (a) ligand **LA** and complex **S1** and (b) ligand **LD** and complex **S2** in CH_3CN (c = 10 μM).

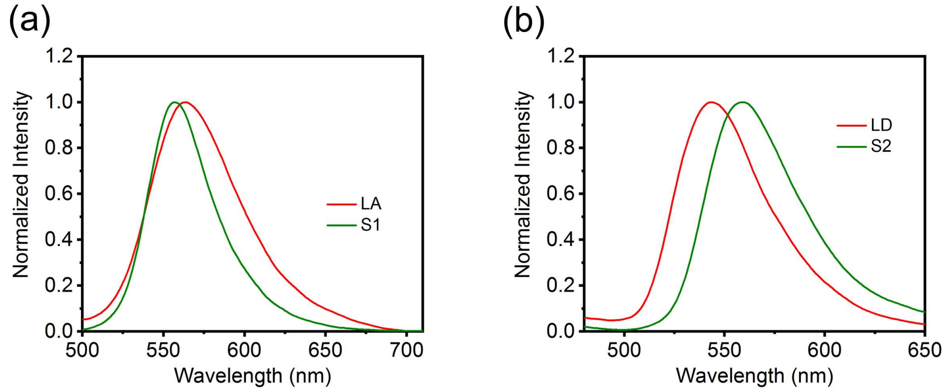

Figure S45. Normalized PL spectra of (a) ligand **LA** and complex **S1** and (b) ligand **LD** and complex **S2** in CH_3CN (c = 10 μM, λ_{ex} = 330 nm).

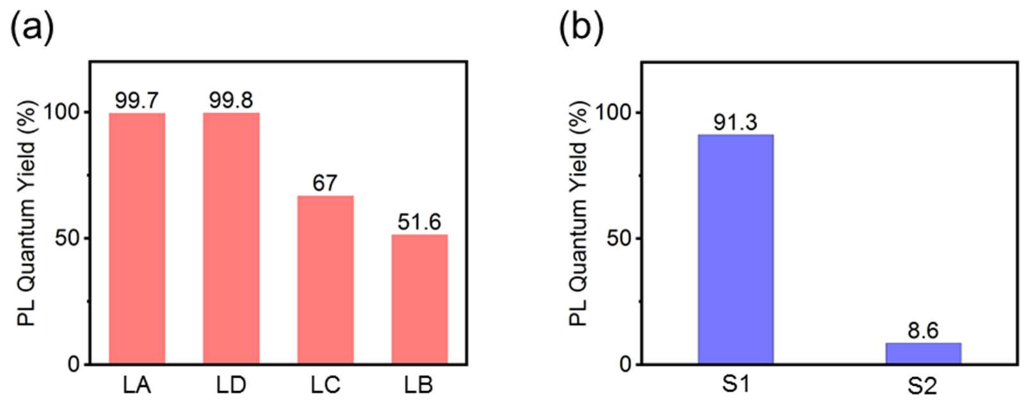

Figure S46. Absolute PL quantum yields of (a) ligands **LA**, **LB**, **LC**, and **LD** in $CHCl_3$ (c = 10 μM) and (b) complexes **S1** and **S2** in CH_3CN (c = 10 μM).

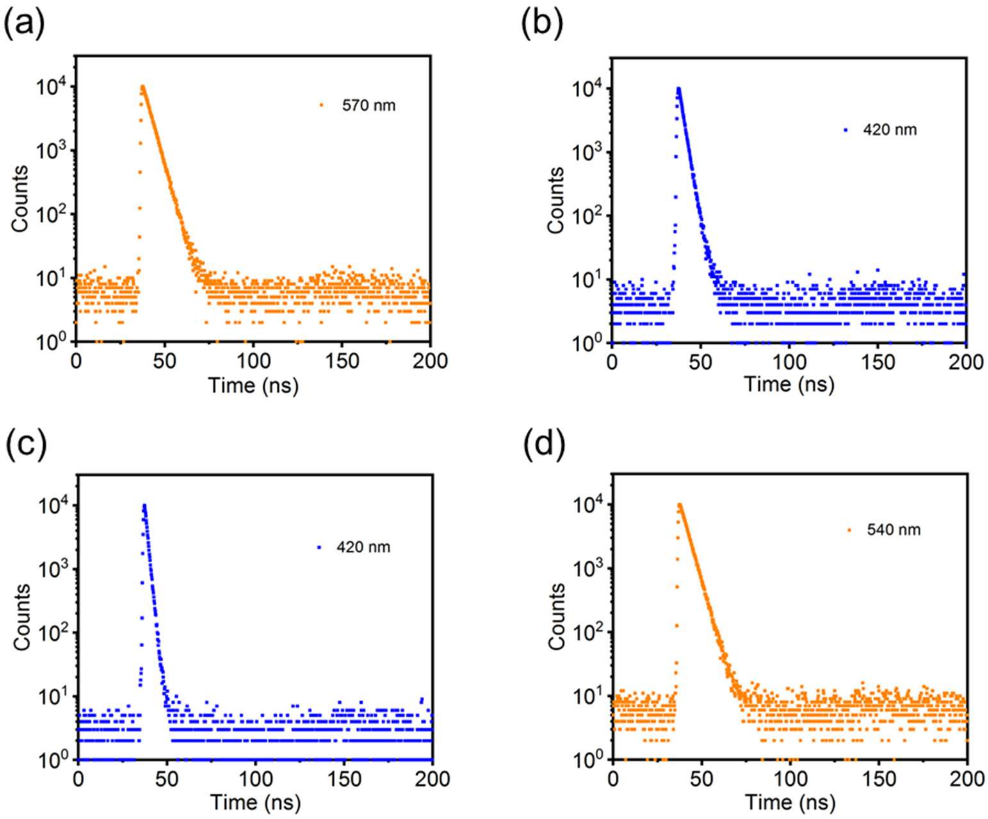

Figure S47. Time-resolved fluorescence decay profiles of ligands (a) **LA** at 570 nm, (b) **LB** at 420 nm, (c) **LC** at 420 nm, and (d) **LD** at 540 nm in $CHCl_3$ (c = 10 μM).

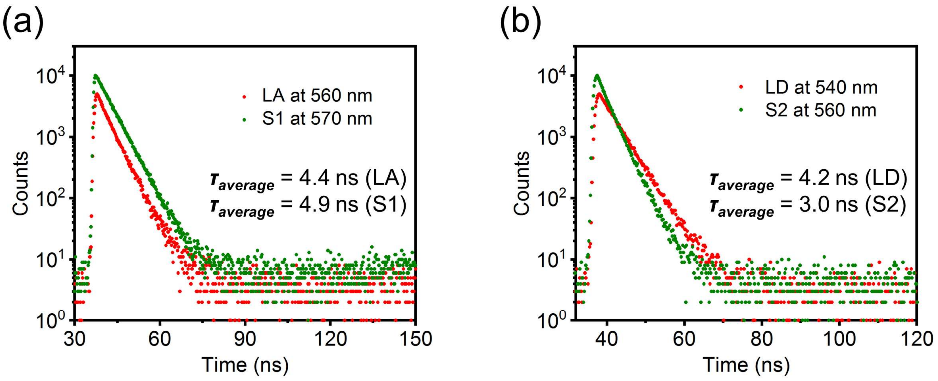

Figure S48. Time-resolved fluorescence decay profiles of (a) ligand **LA** at 560 nm and complex **S1** at 570 nm and (b) ligand **LD** at 540 nm and complex **S2** at 560 nm in CH_3CN (c = 10 μM).

Ligands		τ_1	τ_2	$\tau_{average}$	χ^2	k_r/ $10^7 s^{-1}$	k_{nr}/ $10^7 s^{-1}$
**LA**	Lifetime/ns	4.37	-	4.37	1.25	22.8	0.07
	Percent	100.00	-				
**LB**	Lifetime/ns	2.47	4.70	2.58	1.28	20.0	18.8
	Percent	95.07	4.93				
**LC**	Lifetime/ns	1.53	-	1.53	1.07	43.8	21.6
	Percent	100.00	-				
**LD**	Lifetime/ns	4.42	-	4.42	1.32	22.6	0.05
	Percent	100.00	-				

Table S1. Summary of photophysical properties of ligands **LA**, **LB**, **LC**, and **LD** in $CHCl_3$. k_r = radiactive decay rate (Φ_F/τ); k_{nr} = non-radiative decay rate ($1/\tau - k_r$).

Complexes		τ_1	τ_2	$\tau_{average}$	χ^2	k_r/ $10^7 s^{-1}$	k_{nr}/ $10^7 s^{-1}$
**S1**	Lifetime/ns	4.90	-	4.90	1.27	18.6	1.8
	Percent	100.00	-				
**S2**	Lifetime/ns	1.94	3.24	3.00	1.28	2.9	30.5
	Percent	17.75	82.25				

Table S2. Summary of photophysical properties of complexes **S1** and **S2** in CH_3CN. k_r = radiactive decay rate (Φ_F/τ); k_{nr} = non-radiative decay rate ($1/\tau - k_r$).

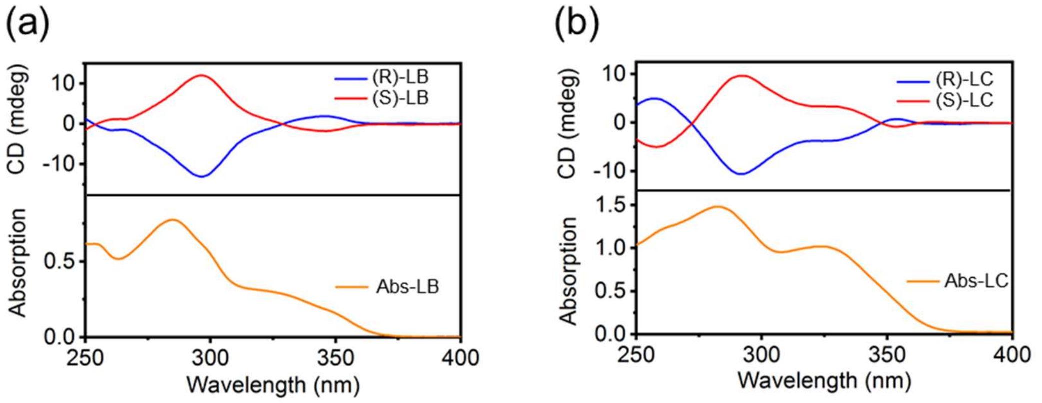

Figure S49. UV/vis absorption (c = 10 μM) and CD spectra (c = 10 μM) of ligands (a) **(R)/(S)-LB** and (b) **(R)/(S)-LC** in $CHCl_3$.

3. Theoretical calculation

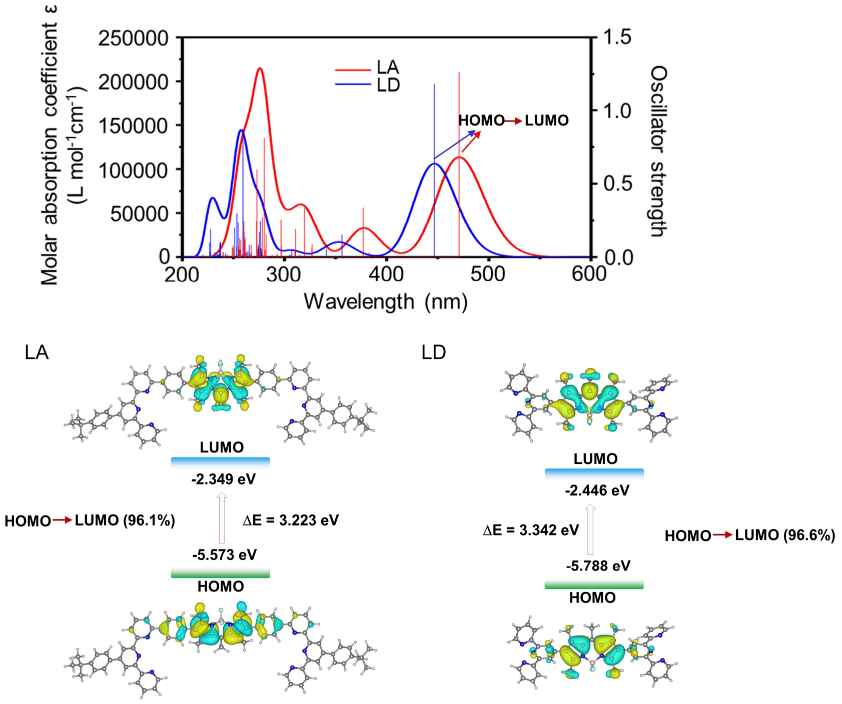

Figure S50. TD-DFT calculated UV/vis absorption spectra and selected molecular orbitals of **LA** and **LD** at the B3LYP/6-31G (d) level of theory in $CHCl_3$.

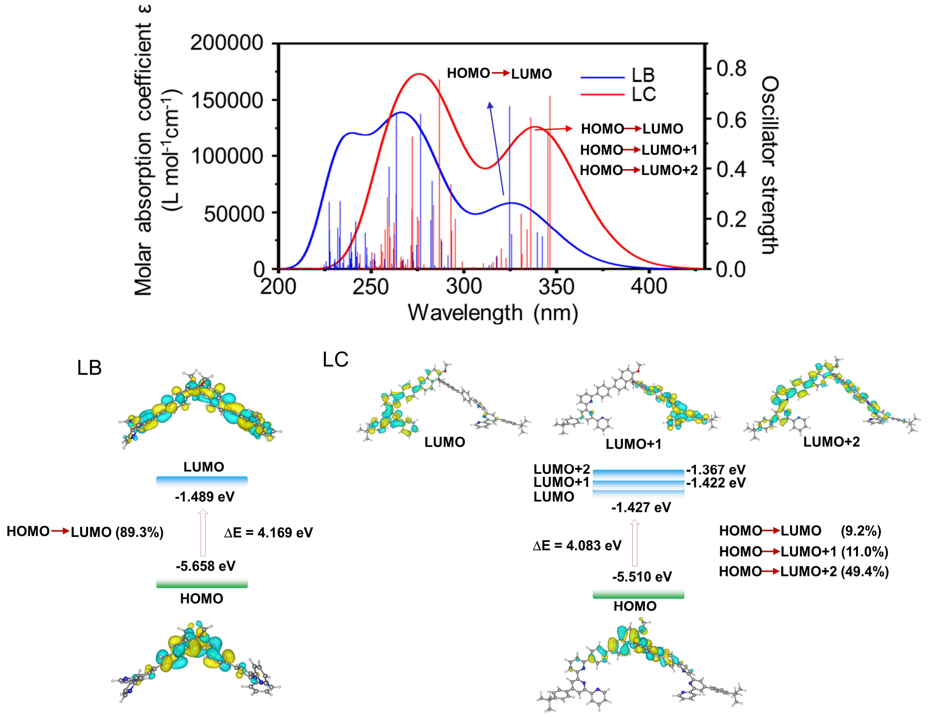

Figure S51. TD-DFT calculated UV/vis absorption spectra and selected molecular orbitals of **LB** and **LC** at the B3LYP/6-31G (d) level of theory in $CHCl_3$.

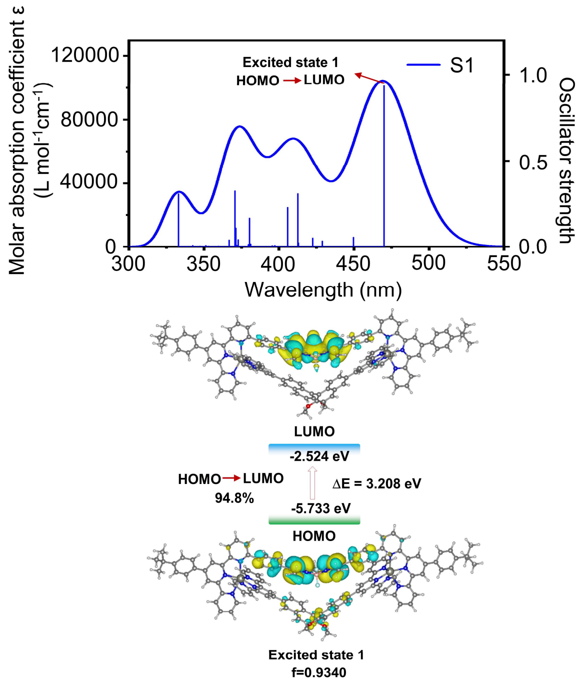

Figure S52. TD-DFT calculated UV/vis absorption spectra and selected molecular orbitals of **S1** at the B3LYP/6-31G (d) level of theory in CH_3CN.

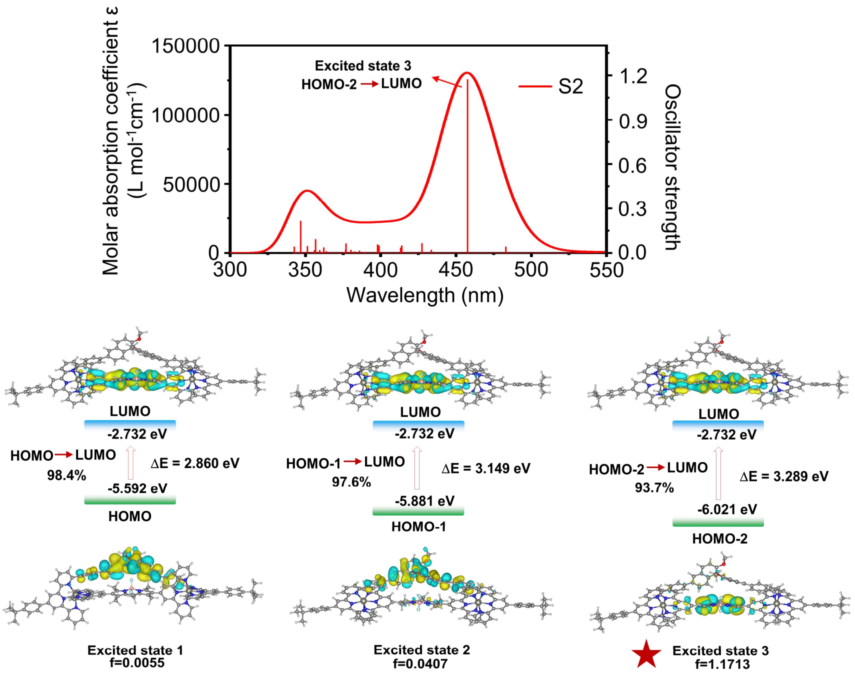

Figure S53. TD-DFT calculated UV/vis absorption spectra and selected molecular orbitals of **S2** at the B3LYP/6-31G(d) level of theory in CH_3CN.

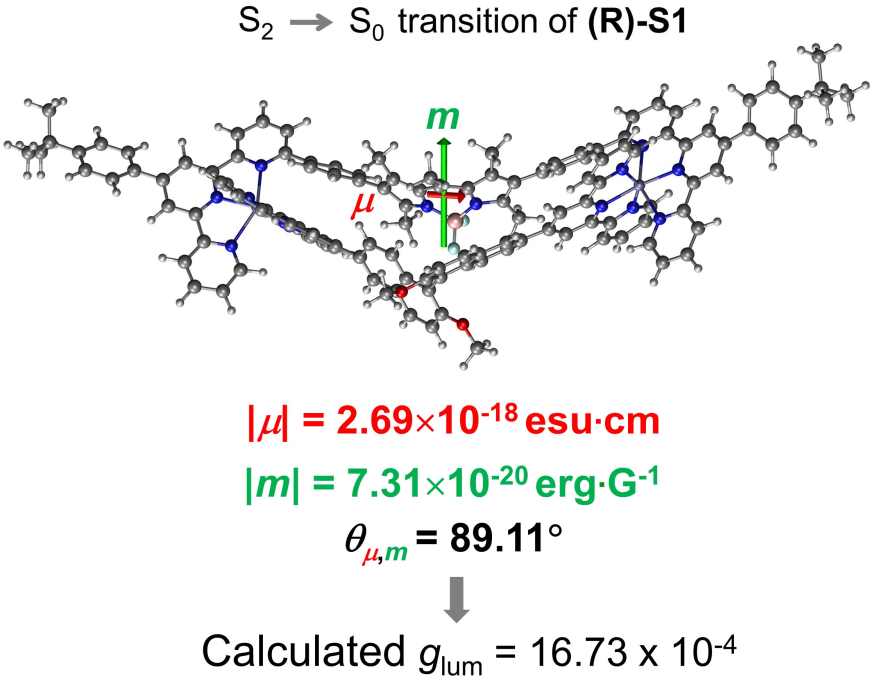

Figure S54. $S_2 \rightarrow S_0$ transition electric and magnetic dipole moments of **(R)-S1**. The transition magnetic dipole moment vector is shown in green and the transition electric dipole moment vector is shown in red.

4. X-ray crystallographic data and structures

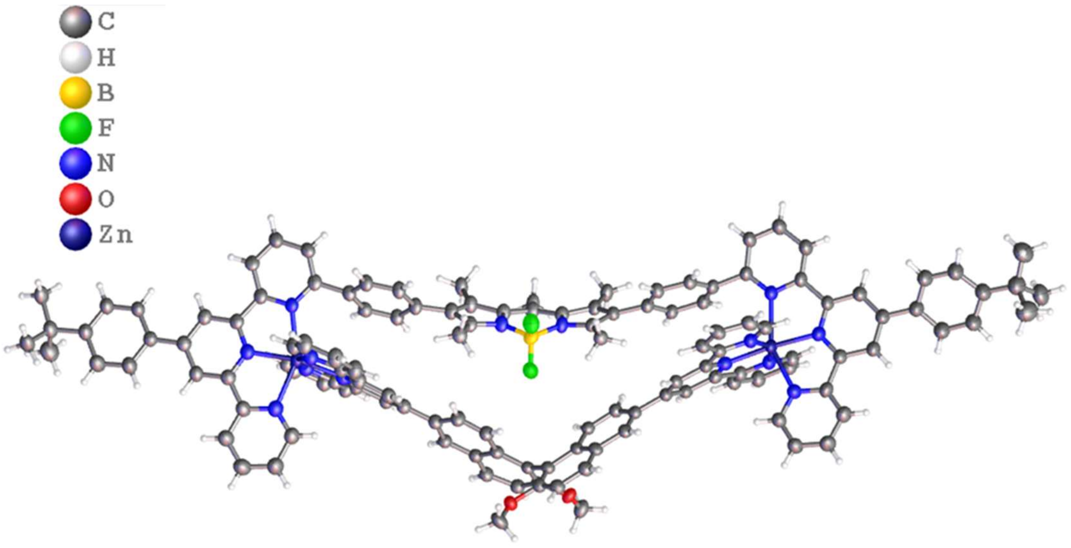

Figure S55. The ORTEP drawing of structure of complex **(R)-S1**.

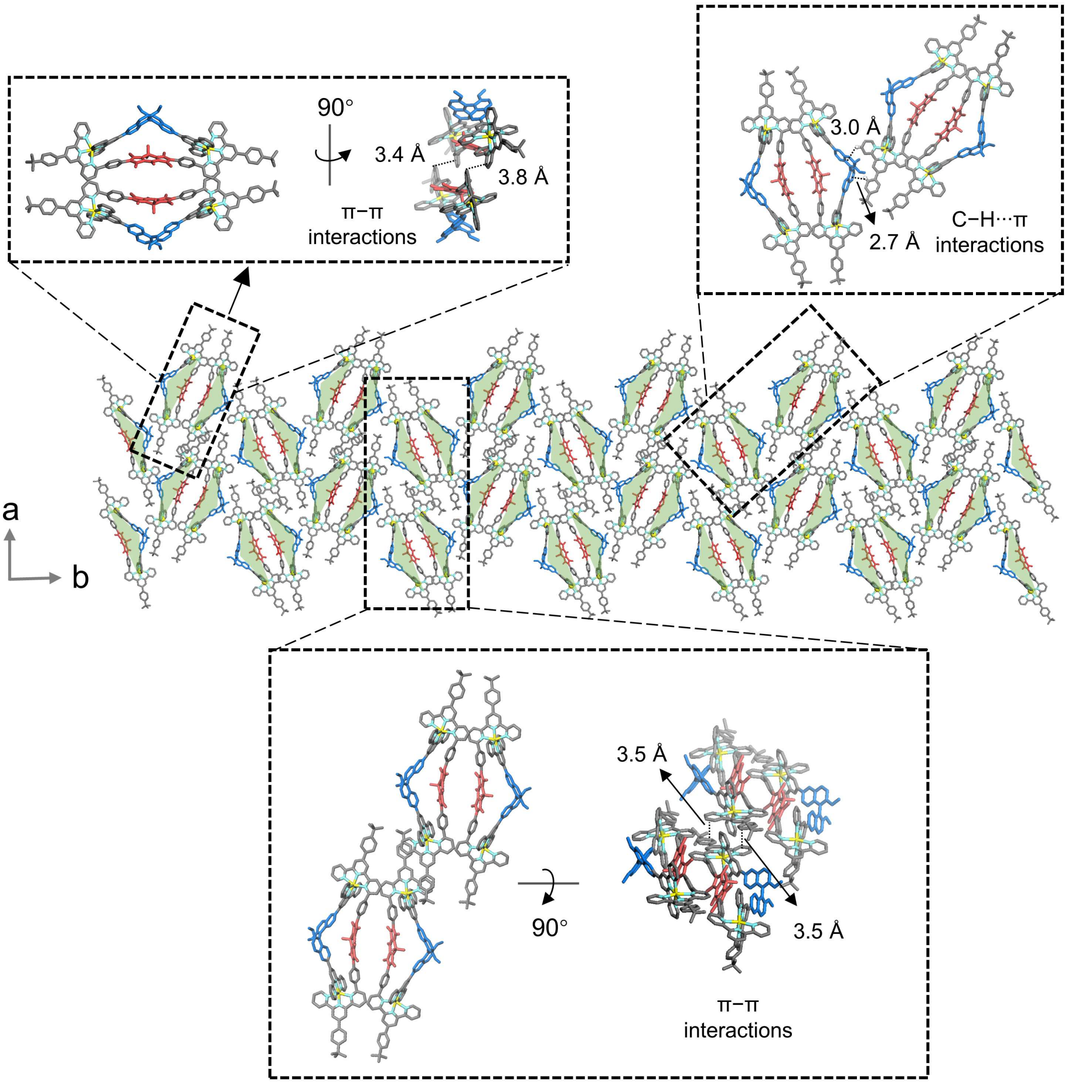

Figure S56. The packing arrangements of complex **R-S1**. All solvent molecules and counterions are omitted for clarity.

Table S3. Crystal data and structure refinement for complex (R)-S1.

Identification code	**(R)-S1**
Empirical formula	$C_{128}H_{103}BF_{26}N_{14}O_2P_4Zn_2$
Formula weight	2624.56
Temperature/K	273.15
Crystal system	triclinic
Space group	$P2_1$
a/Å	13.9352 (16)
b/Å	46.623 (6)
c/Å	22.935 (3)
α/°	90
β/°	102.658 (4)
γ/°	90
Volume/Å^3	14538 (3)
Z	4
ρ_{calc}g/cm^3	1.168
μ/mm^{-1}	0.413
F(000)	5238.0
Crystal size/mm^3	0.2 × 0.15 × 0.14
Radiation	Synchrotron (λ = 0.68883)
2Θ range for data collection/°	1.956 to 48.402
Index ranges	$-16 \le h \le 16$, $-55 \le k \le 55$ $-27 \le l \le 27$
Reflections collected	163239
Independent reflections	48584 [R_{int} = 0.0486, R_{sigma} = 0.0557]
Data/restraints/parameters	48584/17427/3151
Goodness-of-fit on F^2	1.050
Final R indexes [I>=2σ (I)]	R_1 = 0.0599, wR_2 = 0.1636
Final R indexes [all data]	R_1 = 0.0674, wR_2 = 0.1694
Largest diff. peak/hole / e Å^{-3}	1.00/-0.46

5. References

1. Bruker. *APEX3*. Bruker AXS Inc., Madison, Wisconsin, USA, 2018.
2. Bruker. SAINT. Data Reduction Software, 2018.
3. G. M. Sheldrick, *SADABS. Program for Empirical Absorption Correction.* University of Gottingen, Germany, 1996.
4. G. M. Sheldrick, *Acta Cryst.,* 2015, **A71**, 3–8.
5. G. M. Sheldrick, *Acta Cryst.,* 2008, **A64**, 112–122.
6. G. M. Sheldrick, *Acta Cryst.,* 2015, **C71**, 3–8.
7. O. V. Dolomanov, L. J. Bourhis, R. J. Gildea, J. A. K. Howard and H. Puschmann, *J. Appl. Cryst.,* 2009, **42**, 339–341.
8. A. L. Spek, *J. Appl. Cryst*. 2003, **36**, 7–11.
9. Gaussian 16, Revision A.03, M. J. Frisch, G. W. Trucks, H. B. Schlegel, G. E. Scuseria, M. A. Robb, J. R. Cheeseman, G. Scalmani, V. Barone, G. A. Petersson, H. Nakatsuji, X. Li, M. Caricato, A. V. Marenich, J. Bloino, B. G. Janesko, R. Gomperts, B. Mennucci, H. P. Hratchian, J. V. Ortiz, A. F. Izmaylov, J. L. Sonnenberg, D. Williams-Young, F. Ding, F. Lipparini, F. Egidi, J. Goings, B. Peng, A. Petrone, T. Henderson, D. Ranasinghe, V. G. Zakrzewski, J. Gao, N. Rega, G. Zheng, W. Liang, M. Hada, M. Ehara, K. Toyota, R. Fukuda, J. Hasegawa, M. Ishida, T. Nakajima, Y. Honda, O. Kitao, H. Nakai, T. Vreven, K. Throssell, J. A., Jr. Montgomery, J. E. Peralta, F. Ogliaro, M. J. Bearpark, J. J. Heyd, E. N. Brothers, K. N. Kudin, V. N. Staroverov, T. A. Keith, R. Kobayashi, J. Normand, K. Raghavachari, A. P. Rendell, J. C. Burant, S. S. Iyengar, J. Tomasi, M. Cossi, J. M. Millam, M. Klene, C. Adamo, R. Cammi, J. W. Ochterski, R. L. Martin, K. Morokuma, O. Farkas, J. B. Foresman, D. J. Fox, Gaussian, Inc., Wallingford CT, 2016.
10. S. Grimme, J. Antony, S. Ehrlich and H. Krieg, A Consistent and Accurate ab initio Parametrization of Density Functional Dispersion Correction (DFT-D) for the 94 Elements H-Pu, *J. Chem. Phys.*, 2010, **132**, 154104.
11. K. Momma and F. Izumi, "VESTA: a three-dimensional visualization system for electronic and structural analysis," *J. Appl. Crystallogr.*, 2008, **41**, 653–658.
12. T. Lu and F. Chen, *J. Comput. Chem.,* 2012, **33**, 580–592.
13. J. Molec, Graphics 1996, **14.1**, 33–38.
14. N. Han, J. Ma, H. Yu, J. Shi, Q. Bai, X. Jiang, Z. Zhang, P. Wang, J. Yu and M. Wang, Sandwich-like Heterochromophore Metallo-Supramolecules Based on Dense Chromophore Arrangements with Energy and Chirality Transfer Properties, *CCS Chem.*, 2024, **6**, 1264–1277.
15. M. Wang, K. Wang, C. Wang, M. Huang, X.-Q. Hao, M.-Z. Shen, G.-Q. Shi, Z. Zhang, B. Song, A. Cisneros, M.-P. Song, B. Xu and X. Li, Self-Assembly of Concentric Hexagons and Hierarchical Self-Assembly of Supramolecular Metal–Organic Nanoribbons at the Solid/Liquid Interface, *J. Am. Chem. Soc.*, 2016, **138**, 9258–9268.
16. A. M. Durantini, L. E. Greene, R. Lincoln, S. R. Martínez and G. Cosa, Reactive Oxygen Species Mediated Activation of a Dormant Singlet Oxygen Photosensitizer:

From Autocatalytic Singlet Oxygen Amplification to Chemicontrolled Photodynamic Therapy, *J. Am. Chem. Soc.*, 2016, **138**, 1215–1225.
17. M. Kimura, M. Sano, T. Muto, K. Hanabusa and H. Shirai, Self-Assembly of Twisted Bridging Ligands to Helical Coordination Polymers, *Macromolecules* 1999, **32**, 7951–7953.
